# The femtochemistry of nitrobenzene following excitation at 240 nm

**DOI:** 10.1038/s42004-025-01672-2

**Published:** 2025-09-01

**Authors:** Chow-Shing Lam, Tai-Che Chou, Joseph McManus, Ciara Hodgkinson, Michael Burt, Mark Brouard

**Affiliations:** 1 Department of Chemistry, University of Oxford, The Chemistry Research Laboratory, Oxford, UK; 2 Department of Chemistry, Trent University, Peterborough, ON Canada

**Keywords:** Excited states, Photochemistry

## Abstract

Although the photochemistry of nitrobenzene has been extensively studied, the assignment of fragmentation channels and their specific dynamics remains challenging. Here the photochemistry of nitrobenzene following 240 nm excitation into its S_4_ excited singlet state is investigated by femtosecond laser-induced ionization using an intense 800 nm pulse, coupled with time-resolved Coulomb explosion imaging and covariance mapping. We assign photochemical channels by observing correlations between the molecular fragment ions of the associated product pairs, enabling the time-resolved dynamics of channels leading to NO, NO_2_, and C_6_H_5_NO to be fully characterized. NO is produced via two distinct pathways, leading to translationally cold and hot photofragments with risetimes of  ~ 8 ps and  ~ 14 ps, respectively. NO_2_ photofragments are characterised by a bimodal risetime of  ~ 8 ps and  ≳ 2 ns, and can be detected within the first picosecond following ultra-violet photon absorption. C_6_H_5_NO is formed with a risetime of 17 ps. Kinetic energy disposals determined for the three chemical channels agree well with previous work. The techniques employed offer new opportunities to study the time-resolved photochemistry of relatively complex molecules in the gas phase.

**Figure:**
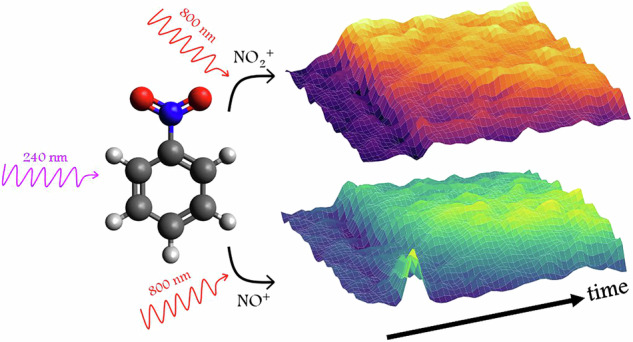

## Abstract (web summary)

Although the photochemistry of nitrobenzene has been extensively studied, the assignment of fragmentation channels and their specific dynamics remains challenging. Here, the authors combine femtosecond laser-induced ionization, Coulomb explosion imaging and covariance mapping to fully characterize the time-resolved dynamics of fragmentation channels leading to the formation of NO_2_, NO and C_6_H_5_NO.

## Introduction

The gas phase photochemistry of nitrobenzene has attracted considerable attention over recent years, arising from its important role as a pollutant in the atmosphere^1^ and as a model system for energetic materials^2^. Nitrobenzene exhibits a rich and complex photochemistry, which has been studied extensively, both in the gas phase and in solution^3,4^. A particular focus has arisen from the fact that nitroaromatic molecules can release NO on photoexcitation.

Numerous experimental techniques have been used to investigate the photochemistry of nitrobenzene^5–12^, and much of the gas phase work has been the subject of recent review^3,4^. The main dissociation pathways, and their wavelength dependence, have been established as follows^5,6,8^:

1 $${{{\rm{C}}}}_{6}{{{\rm{H}}}}_{5}{{{\rm{NO}}}}_{2}+h\nu \longrightarrow {{{\rm{C}}}}_{6}{{{\rm{H}}}}_{5}+{{{\rm{NO}}}}_{2}\quad\quad\quad\quad\quad\quad\quad\quad\quad$$

2 $$\longrightarrow {{{\rm{C}}}}_{6}{{{\rm{H}}}}_{5}{{\rm{O}}}+{{\rm{NO}}} ({{\rm{a}}})$$

$$\,\,\,\,\,\,\,\,\,\,\,\,\,\,\,\,\,\,\,\,\,\,\,\,\,\,\,\,\,\--\to {{{\rm{C}}}}_{5}{{{\rm{H}}}}_{5}+{{\rm{CO}}}+{{\rm{NO}}}\quad ({{\rm{b}}})$$

3 $$\longrightarrow {{{\rm{C}}}}_{6}{{{\rm{H}}}}_{5}{{\rm{NO}}}+{{\rm{O}}}\quad\quad$$

with Channel (1), the formation of NO_2_, being dominant at wavelengths close to those employed in the present study (240 nm). Channel (3) is believed to play a minor role, with a yield of  ~ 3% at wavelengths near 240 nm^5^. Channel (2) leads to two sets of products, the first (a) leading to C_6_H_5_O fragments, the second (b) involving subsequent dissociation of energized C_6_H_5_O to C_5_H_5_ and CO on timescales uncharacterized in the nanosecond pump-probe experiments^5,6,8^. In addition, NO is formed with a bimodal velocity distribution, associated with at least two dissociation pathways^5,6,8,9^, assigned by Suits et al. to a fast, triplet channel, and a slow, roaming mechanism^9^. Time-resolved ultrafast electron diffraction (UED)^7,12^ and time-resolved photoelectron imaging (TRPEI)^10,11^ studies present somewhat different pictures for the relaxation and dissociation timescales. Nevertheless, they generally reveal that, following UV excitation to high-lying singlet states, rapid internal conversion (IC) to either S_1_ or S_0_ occurs within tens of femtoseconds, with longer timescales (hundreds of femtoseconds) associated with S_1_  → T_1_ intersystem crossing (ISC), and tens to hundreds of picoseconds for ISC from T_1_ back to S_0_^11^.

Considerable effort has also been devoted to computational investigations into the photochemistry of nitrobenzene and related systems^13–20^. Photon absorption at 240 nm excites nitrobenzene to the ${{{{\rm{S}}}}_{4}}^{1}({L}_{a}{\pi \pi }^{* })$ electronically excited singlet state, which is a charge transfer state associated with excitation from the benzene ring to the nitro group^16–20^. The more recent theoretical work^17–20^ provides general support for the electronic relaxation mechanism outlined above, in particularly providing potential pathways for NO_2_ production via the triplet ^3^(*n*_B_, π*) state following ISC from S_1_ or higher lying states, and NO production from S_0_ following either ISC from T_1_^3^(*n*_A_, π*) or the triplet ^3^(*n*_B_, π*) state^17,20^.

In this work we take a different experimental approach to those employed previously in the study of nitrobenzene by probing the time-resolved dynamics via a combination of femtosecond laser-induced ionization using an intense, near-infrared (NIR) laser pulse, coupled with Coulomb explosion imaging (CEI) and covariance mapping methods^21–23^. Such experiments have been shown to be sensitive to the nuclear dynamics^24^, and should provide complementary information to TRPEI and UED. Here we demonstrate the application to a relatively complex gas phase molecule, under conditions in which molecular fragment ions originating from synchronously generated product pairs can be observed in time-resolved covariance. We show that making such measurements on the timescales of molecular photodissociation enables the unambiguous assignment of fragmentation channels and the determination of channel specific dynamics, such as time constants and momentum disposals.

Following excitation of nitrobenzene with an ultra-violet (UV) femtosecond pulse at 240 nm (95 fs, peak intensity 4 × 10^12^ W cm^−2^), the fragmenting excited parent molecule is multiply-ionized to a variety of charged states using a non-resonant 800 nm NIR laser pulse (42 fs, 2 × 10^14^ W cm^−2^). The momenta of the ions thereby generated are recorded using velocity-map ion imaging (VMI)^25,26^ and a fast time-stamping camera (see “Methods”), which enables the correlated momenta of multiple ions to be detected simultaneously in the same time-of-flight cycle.

## Results and discussion

### Time-resolved momentum distributions

**Fig. 1:**
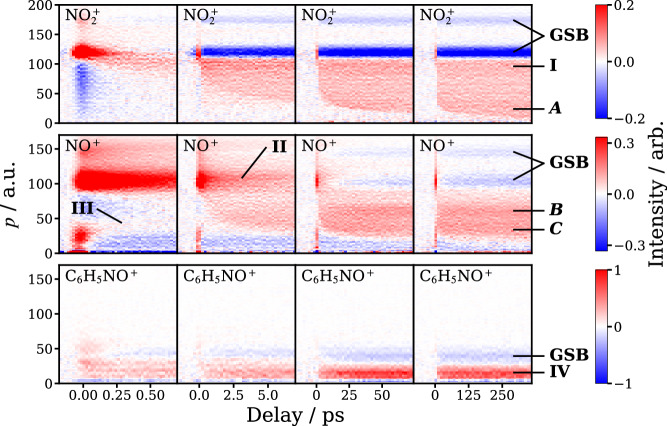
Time-resolved momentum distributions. Time-resolved momentum distributions for the fragment ions ${{{\rm{NO}}}}_{2}^{\,+}$ (top row), NO^+^ (middle), and C_6_H_5_NO^+^ (bottom) following UV excitation and NIR probing of nitrobenzene. The columns correspond to the four different delay scan ranges, spanning (from left to right) 1 ps, 10 ps, 100 ps, and 500 ps. The data were obtained by subtracting the average signal from Δ*t* < 0 from the momentum distributions extracted from Abel-inversion of the raw time-dependent velocity-map ion images, examples of which are shown in the Supplementary Note 2. Red data are positive, signifying enhancement of signal following UV excitation, blue data are negative, signifying signal depletion (ground state bleaching, labeled GSB). Various features in the momentum distributions are labeled and referred to in the main text.

Experiments were performed under a regime in which ionization of species by the UV laser alone was at least an order of magnitude less likely than with the NIR pulse. However, importantly, as noted above, the intensity of the latter was considerably less than that required for atomization of the parent molecule, and significant amounts of charged molecular fragments were detected in the experiment, including the ions associated with the primary photofragments arising from Channels (1)–(3) (see Supplementary Note 1). The ion yields and ion images suggest that the dissociating molecules we observe typically acquire 1 to 3 positive charges during the NIR laser pulse. Figure 1 presents the time-resolved momentum distributions for ${{{\rm{NO}}}}_{2}^{\,+}$, NO^+^ and C_6_H_5_NO^ +^ associated with the primary photochemical Channels (1)–(3), respectively. Data were recorded over four delay-scan ranges, spanning 1 ps (left column), 10 ps, 100 ps, and 500 ps (right column). The time-delay, Δ*t*, measures the arrival time of the NIR probe pulse minus that for the UV pump pulse, such that positive delays correspond to the UV pulse arriving first. In Fig. 1, we highlight the changes in the ion momentum distribution induced by the UV pulse by subtracting off the average signal before *t*_0_ (i.e., when Δ*t* < 0), which is dominated by the NIR probe-only signal, from subsequent data. Features in red therefore correspond to signal enhancement by absorption of the UV pulse, whilst those in blue correspond to depletion. The time-resolved momentum distributions were obtained from the time-resolved ion images via Abel inversion of the raw ion image data (see “Methods” and Supplementary Note 2).

#### Constant momentum features

There are several qualitative aspects of the time-resolved momentum distributions that are noteworthy. At short delay times (the left hand column in Fig. 1), we see an enhancement of signal for several species that closely follows the 97 fs cross-correlation width of the UV and NIR laser pulses. We associate these signals with multiphoton ionization by the 800 nm pulse, which is enhanced when the UV pulse is present. The UV pulse excites nitrobenzene to the S_4_ state, from where the efficiency of 800 nm multiphoton ionization is enhanced compared to that for ground state molecule, and quickly drops when the delay goes beyond *t*_0_ and the system migrates out of the Franck-Condon region.

A number of features (labeled I to IV in Fig. 1) are roughly constant in momentum with time delay. Features labeled I and II are associated with NIR ionization of the intact UV-excited parent molecule or parent ion, followed by their subsequent fragmentation into a number of species, including the detected fragment ions. Note that in this case we see evidence of the detected ion partnered by both neutral, singly charged, and (to a minor extent) doubly charged cofragments, with a corresponding increase in the kinetic energy and hence momentum of the detected ion (Supplementary Note 3). Other momentum-invariant features arise from the generation of a neutral fragment pair following UV photoabsorption, and subsequent ionization of only one of the fragments by the NIR probe (e.g., the features labeled III and IV in Fig. 1). The latter features generally increase in intensity over time, reflecting the timescales for dissociation into one of the neutral Channels (1)–(3) in the UV-induced fragmentation process, whilst the former are usually either constant or decrease in intensity with time, in the latter case reflecting the electronic relaxation and eventual dissociation of the parent molecule. The quantitative analysis of the time constants extracted from the experiments is discussed further in Section “Kinetic data and simulations”, and in the Supplementary Note 5.

The interpretation of features I and II in Fig. 1 requires an understanding of how the NIR ionization efficiency of nitrobenzene varies with electronic and vibrational state, which is currently unknown. Whilst these features are likely to contain interesting information about the lifetime of the parent molecule in different electronic states at early times, in the present work we have not attempted their quantitative analysis. Nevertheless, it is interesting to note that some of these features decay on time-scales of a few hundred femtoseconds, whilst others (e.g., feature II) decay on tens of picosecond timescales, consistent with the operation of both singlet and triplet pathways in the photochemistry of nitrobenzene (see further below and Supplementary Note 5).

#### Coulomb curves

Our principal focus is the features that decrease in momentum as a function of time delay. These are most evident for the NO_2_ and NO photofragments (labeled *A*, *B* and *C* in Fig. 1), but are also observed in the momentum distributions of other photofragments. The decrease in momentum with time delay is associated with neutral photodissociation of the parent molecule, followed by NIR ionization of both neutral photofragments as they separate. The resulting time-resolved momentum distributions are referred to as “Coulomb curves”^27^, reflecting the diminishing Coulomb repulsion experienced by a pair of charged species generated at increasing separations. In the present experiments, we generally only see Coulomb curves associated with singly charged photofragment pairs. We find that the curves are broadened compared with those observed in simpler systems, such as found in the methyl halides^28,29^, because the fragments generated in the photodissociation of nitrobenzene are born with broad momentum distributions, over a wide range of delay times, as confirmed by the simulations presented in Section “Kinetic data and simulations”.

**Fig. 2:**
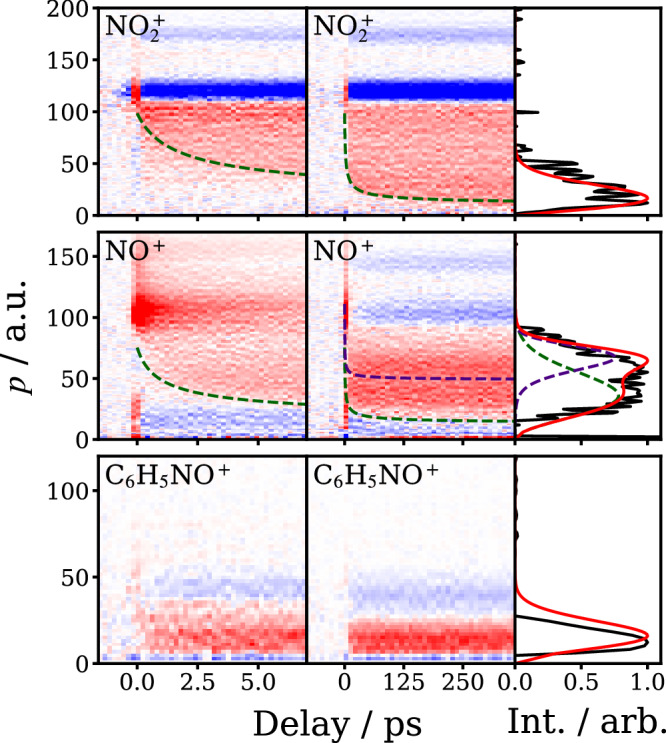
Coulomb curves and asymptotic momentum distributions. Left and middle columns: time-resolved momentum distributions for ${{{\rm{NO}}}}_{2}^{+}$ (top row), NO^+^ (middle row) and C_6_H_5_NO^+^ (bottom row) ionized by the NIR probe laser at 800 nm, and associated with Channels (1)–(3), respectively. The dark dashed lines shown in the left and middle panels of the top two rows highlight the Coulomb curves for the NO_2_ and NO photofragments. Note that NO is formed with two distinct momentum components, a slow, prompt component (highlighted in the left panel) and a fast, delayed component (highlighted in the middle panel), in agreement with previous work^5,6,8,9^. The right panels show comparisons of the long-time momentum distributions obtain in this work at 240 nm (black lines) with those derived from previous work at 248 nm by Lin et al. (red lines)^8^.

The ${{{\rm{NO}}}}_{2}^{\,+}$ and NO^+^ Coulomb curves are highlighted in Fig. 2. ${{{\rm{NO}}}}_{2}^{\,+}$ displays a single Coulomb curve, suggesting neutral NO_2_ photofragments (Channel (1)) are generated via a single dominant mechanism on the timescales shown. Note that the Coulomb curve is overlapped with the time-invariant feature labeled I in Fig. 1, which was isolated using the procedure described in detail in the Supplementary Note 3. At long delay times, when the C_6_H_5_ and NO_2_ photofragments are well-separated, ionization of both species by the NIR pulse leads to minimal Coulomb repulsion. Therefore, the asymptotic long-time momentum distribution of ${{{\rm{NO}}}}_{2}^{\,+}$ associated with the Coulomb curve (obtained by integrating the long time delay data over the final 100 ps) reflects the momentum distribution of the nascent NO_2_ photofragment produced in the UV photodissociation of nitrobenzene. This distribution is compared with that derived from the total kinetic energy release distribution measured previously in nanosecond pump-probe experiments^8^ conducted at 248 nm (see the right panels of Fig. 2). The present data are in reasonable agreement with previous work. A similar analysis of the long time delay (300–400 ps) NO^+^ Coulomb curves reveals a bimodal NO velocity distribution, with fast and slow components, again consistent with previous work^5,6,8,9^, and assigned by Suits et al. respectively to triplet (fast) and roaming (slow) dissociation mechanisms^9^. Unlike in previous studies, the present work additionally reveals that the fast NO channel is delayed with respect to the slow NO channel, as is clearly visible from the data shown in Figs. 1 and 2 (see also Section “ Kinetic data and simulations”).

Channel (3) is observed exclusively through signals associated with ionization of the molecular fragment alone, C_6_H_5_NO (feature IV in Fig. 1), and hence a Coulomb curve is not observed for this channel. Under the present conditions, it would appear that the O atom cofragment is ionized relatively inefficiently. Note that the momentum distribution associated with C_6_H_5_NO^ +^, shown in Fig. 2, is in good agreement with that observed previously for the Channel (3)^8^, lending support to our interpretation of the time-resolved C_6_H_5_NO^ +^ data.

### Covariance data

**Fig. 3:**
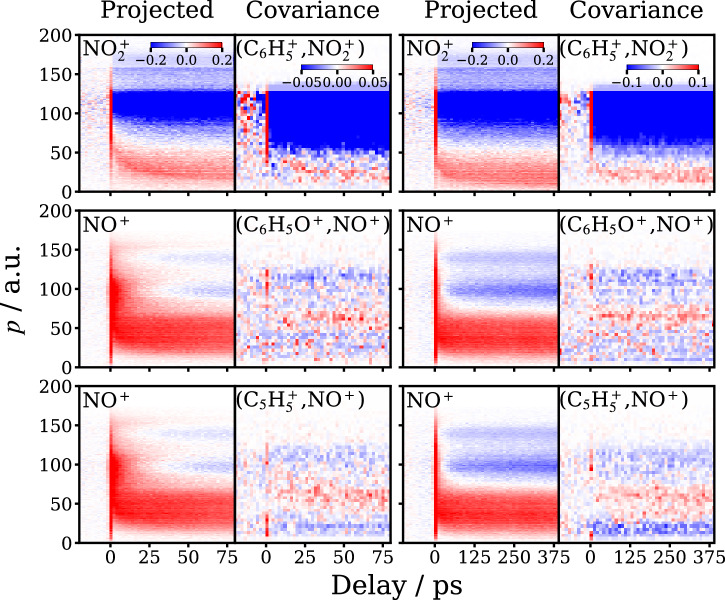
Time-resolved covariant photofragment momentum distributions. First and third columns: time-resolved projected momentum distributions for the photofragments indicated. Second and fourth columns: time-resolved projected momentum distributions obtained in two-fold covariance, involving the species indicated. The data in the two left hand columns is over a 100 ps range, whilst that in the two right hand panels is on a 500 ps timescale. Note that here cov(*X*, *Y*) is plotted as a function of the momentum of *Y*. The data have been obtained by subtracting the average covariance data from Δ*t* < 0 from the projected covariant momentum distributions extracted from the analysis. The color scaling runs from +1 (red) to −1 (blue) unless otherwise indicated.

Assignment of the NO_2_ and NO photofragment Coulomb curves to specific channels has been confirmed by two-fold covariance analysis, a method for measuring the statistical correlation between fragment ion pairs (see Section “Covariance mapping” and Supplementary Note 4), as illustrated in Fig. 3. Note that here the covariance (*X*, *Y*) is plotted as a function of the momentum of *Y*. The analysis reveals that specific Coulomb curve features can be associated with specific fragment pairs, and hence identifiable photofragment channels.

${{{\rm{NO}}}}_{2}^{\,+}$ is observed in covariance with both ${{{\rm{C}}}}_{6}{{{\rm{H}}}}_{5}^{\,+}$ and ${{{\rm{C}}}}_{6}{{{\rm{H}}}}_{3}^{\,+}$, reflecting the fact that ${{{\rm{C}}}}_{6}{{{\rm{H}}}}_{5}^{\,+}$ is unstable under the conditions of the present experiments, and can lose neutral H_2_ or 2H on the detection timescales. This leads to a slight broadening of the covariance images and momentum of ${{{\rm{C}}}}_{6}{{{\rm{H}}}}_{3}^{\,+}$ with respect to NO_2_, compared to those for (${{{\rm{C}}}}_{6}{{{\rm{H}}}}_{5}^{\,+}$, NO_2_), which in the latter case are purely back-to-back, as required by momentum conservation^30^ (see Supplementary Note 4.).

NO^+^ is observed clearly in covariance with C_6_H_5_O^+^, ${{{\rm{C}}}}_{6}{{{\rm{H}}}}_{5}^{\,+}$ and ${{{\rm{C}}}}_{5}{{{\rm{H}}}}_{5}^{\,+}$ (see Fig. 3 and Supplementary Note 4). The relatively weak (C_6_H_5_O^+^, NO^ +^) covariance is back-to-back as expected, and is associated with a relatively high momenta compared with that for NO. The covariant (C${}_{5}{{{\rm{H}}}}_{5}^{+}$, NO^ +^) momenta display a broader distribution of momenta, consistent with the translationally cold, internally hot C_6_H_5_O photofragments undergoing secondary dissociation via Channel (2)(b) to C_5_H_5_ + CO. In principle, the time-dependence of the three-fold covariance between the ions of the three photofragments (i.e., ${{{\rm{C}}}}_{5}{{{\rm{H}}}}_{5}^{\,+}$, CO^+^, and NO^+^) formed in Channel (2)(b) would help confirm this assignment, but under the current experimental conditions we were unable to detect three-fold covariance with sufficient statistics to definitively address this point. Further discussion of the covariant momentum distributions is given in the Supplementary Note 4.3.

As noted above, the C_6_H_5_NO + O channel is dominated by signals associated with ionization of the molecular fragment alone, and, consistent with this observation, no clear (C_6_H_5_NO^ +^, O^+^) covariance associated with Channel (3) was observed under the conditions of the present experiments.

### Kinetic data and simulations

**Fig. 4:**
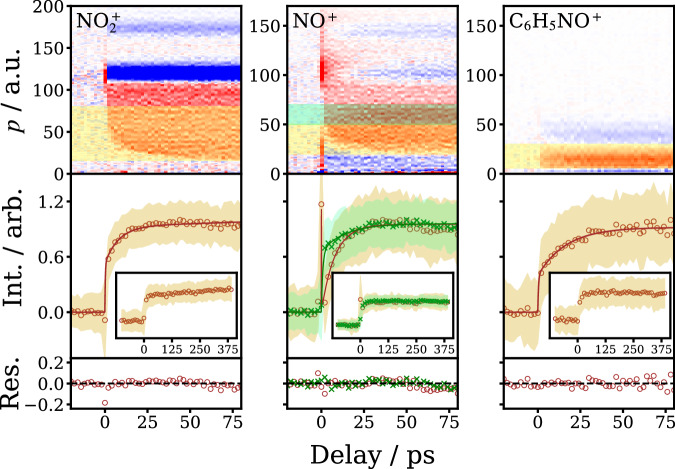
Fits to time-resolved momentum features. Example kinetic data used to extract the time constants given in Table 1. The top row shows the time-resolved momentum distributions for ${{{\rm{NO}}}}_{2}^{+}$ (left), NO^+^ (middle), and C_6_H_5_NO^+^ (right) on a 100 ps timescale, with the regions integrated to obtain the time-resolved intensity data highlighted in different colors. The time-resolved integrated intensities are plotted in the middle panels along with the fits to the data, with the residuals shown in the lower panels. The fits are obtained using the procedures detailed in the main text and Supplementary Note 5. The shaded regions in the middle panels represent the standard deviations of the experimental data points. The insets in the middle panels show the kinetic data on a 500 ps timescale, highlighting the long-time nanosecond growth of signal for ${{{\rm{NO}}}}_{2}^{+}$, but not NO^+^ or C_6_H_5_NO^+^.

The Coulomb curves associated with both NO_2_ and NO, as well as feature IV associated with the C_6_H_5_NO photofragment, increase in intensity as a function of delay-time, reflecting the timescales for UV dissociation of the parent molecule into Channels (1)–(3). NO_2_ photofragments are observable within the first picosecond, as clearly seen in Fig. 1. Fits to the intensity as a function of time, together with simulation, reveal a bimodal distribution for the NO_2_ rise-time, with time-constants of 8 ps and  ≳2 ns (see Fig. 4, “Methods” and Supplementary Note 5). The kinetic data are collected in Table 1. The long time component for NO_2_, *τ*_3_, is consistent with previous experimental observations and RRKM calculations for dissociation of nitrobenzene on the ground electronic state by Lin et al.^8^. Similar analysis of the two Coulomb curves associated with NO^+^ yield rise times, *τ*_2_, of 8 ps (slow NO channel) and 14 ps (fast channel): the fast NO channel is thus delayed with respect to the prompt, slow NO channel (Figs. 2 and 4). Given the similarity of the 8 ps component of the NO_2_ and slow NO photofragments, it is tempting to associate them with a common dissociation pathway, as would be consistent with the latest theoretical calculations from Worth et al. which indicate the common involvement of the triplet ^3^(*n*_B_, π*) electronic state^20^.

**Table 1:** Kinetic data obtained from fits to the time-resolved momentum distributions

Time constant/ps	*τ*_1_	*τ*_2_	*τ*_3_
(Amplitude/%)	(*A*_1_)	(*A*_2_)	(*A*_3_)
Channel 1 (A)	0.25 ± 0.05	8.1 ± 1.2	>2000
	(57 ± 3)	(43 ± 4)	(N.A.)
Channel 2 (B—delayed/fast)	0.87 ± 0.10	14.2 ± 2.4	
	(69 ± 3)	(31 ± 4)	
Channel 2 (C—prompt/slow)	0.14 ± 0.02	7.9 ± 0.4	
	(67 ± 3)	(33 ± 1)	
Channel 3 (IV)	0.42 ± 0.06	16.5 ± 1.4	
	(43 ± 1)	(57 ± 4)	

Time constants and amplitudes associated with the dissociation channels indicated, which are also labeled according to the momentum assignments shown in Fig. 1. Example fits to the data are shown in Fig. 4. Errors represent 2*σ* arising from the fits. The fitting procedures employed are described further in the text and Supplementary Note 5.

**Fig. 5:**
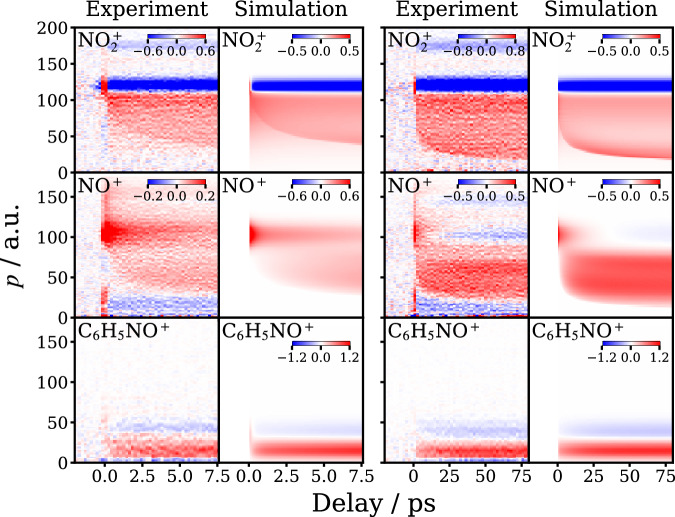
Simulations of the time-resolved momentum distributions. Comparisons of the experimental (columns 1 and 3) and simulated (columns 2 and 4) time-dependent momentum distributions for the ${{{\rm{NO}}}}_{2}^{+}$, NO^+^ photofragments and C_6_H_5_NO^+^ (top to bottom rows, as labeled) over time-ranges of 10 ps (columns 1 and 2) and 100 ps (columns 3 and 4). The color scaling is shown in the bars on each panel except in the cases for which the scaling goes +1 (red) to −1 (blue). The simulations, discussed in more detail in the main text and Supplementary Note 6, use the asymptotic long delay-time momentum distributions shown in Fig. 2 and the kinetic parameters in Table 1.

The kinetic data shown in Table 1 have been used to simulate the time-dependent ion momentum distributions associated with the Coulomb curves shown in Fig. 1. Because the molecular photofragments are produced with a wide range of momenta on the same timescale as the present measurements, modified simulations procedures were required compared with those undertaken previously^28^, as described in detail in Supplementary Note 6. In addition to the kinetic data, the simulations employed the channel momenta derived from the long-time asymptotic distributions observed experimentally, i.e., it was assumed that the neutral photofragment momentum distributions were constant over time. The simulated NO^+^ or ${{{\rm{NO}}}}_{2}^{\,+}$ momenta were determined based on these measured nascent neutral momentum distributions, together with those associated with the Coulomb repulsion between the charged fragment pair at a given time delay determined by NIR ionization. As shown in Fig. 5, the results of the simulations are in good qualitative agreement with the experimental data, supporting the picture of the photofragmentation dynamics presented here.

### Comparisons with previous time-resolved work

We have already compared the photofragment momentum distributions derived from the present work with previous nanosecond experiments^5,6,8,9^. Given the differences in wavelengths employed in these various studies, the agreement between them is reasonable, and the picture of the photochemistry of nitrobenzene that emerges in terms of product channels, branching ratios and kinetic energy releases is largely consistent. Of the time-resolved work^7,10–12^, the most recent TRPEI study by Townsend et al. at 267 nm reported timescales of ≤30 fs, 160–190 fs, and 90–160 ps, which they associated with rapid IC from S_4_ and S_3_ to S_1_ or S_0_, ISC from S_1_ to T_1_, and ISC from T_1_ to S_0_, respectively. Given the time-resolution of the present experiments (~100 fs) we are unable to observe with confidence the shortest of these timescales, but we do see several features in the time-resolved momentum distributions that are broadly consistent with the second of these timescales of 160–190 fs, for example, see *τ*_1_ for the NO_2_ and the prompt/slow NO photofragments (Table 1). The longest decay observed in the TRPEI study is significantly longer than most of the dissociation time constants we measure, but might be consistent with tens of picosecond risetimes we observe for NO_2_ and NO, taking into account the shorter wavelength of the present study. Note that the TRPEI measurements do not observe the long, nanosecond time constant that we see for NO_2_ production, which we associated with unimolecular decay on the ground electronic state of nitrobenzene.

Comparison with the time-resolved UED experiments^7,12^ is also of considerable interest. The first of these studies by Zewail et al., again using 267 nm photoexcitation, found that formation of NO and C_6_H_5_O occurred on an 8.8 ± 2.2 ps timescale. Allowing for the differences in excitation wavelength, this is in reasonable accord with the timescale observed here for the slow, prompt NO photoproducts. Note, however, that the UED study was unable to distinguish the two decay pathways to form translationally hot and cold NO that has been observed in the present work. Furthermore, the earlier UED study found that NO formation accounted for the dominant channel, at least on the picosecond timescale, in apparent contrast to the findings of the nanosecond experiments^5,8^. The present experiments provide a plausible explanation for this discrepancy, namely that NO is formed on the picosecond timescale, whereas NO_2_ is formed predominantly on the nanosecond timescale, albeit with a fast, picosecond minor component. Our simulations based on the present kinetic data, together with the previously measured product yields^5,8^, suggest that NO is likely to appear as the dominant channel at short delay times (a few ps), because of its fast risetime, but as a minor channel relative to NO_2_ at long time (a few ns).

More recently, Wolf et al. have reported a timescale of 160 fs for ground state recovery, using 267 nm excitation and mega-electron volt UED, consistent with the second of the timescales observed by Townsend et al.^11^. However, they did not observe significant photofragment formation within the first 5 ps, an observation at variance with the present experiments. Notwithstanding the differences in UV wavelengths employed, the fact that we clearly see formation of both NO and NO_2_ photofragments within the first 5 ps implies that the UED experiments were less sensitive to signatures of the photofragments than the present CEI experiments.

## Conclusion

We have shown that it is possible to use time-resolved CEI and covariance mapping techniques to characterize molecular photofragment pairs generated during the UV photodissociation of nitrobenzene. The method enables unambiguous channel assignment and dynamical characterization of channels leading to molecular photofragments, which tend to be ubiquitous in photochemistry of larger molecules in the gas phase. In the specific case of nitrobenzene, we find that NO is produced via two distinct pathways, leading to (1) slow, prompt versus (2) fast, delayed NO photofragments with risetimes of  ~ 8 ps and  ~ 14 ps, respectively. NO_2_ is formed with bimodal risetimes of  ~ 8 ps and  ≳2 ns, whilst C_6_H_5_NO appear on a 17 ps timescale. Unlike in recent diffraction studies^7,12^, we show here that all product channels can be readily detected within the first few picoseconds following UV photon absorption. Kinetic energy release data, derived from the measured momentum distributions, are found to be in good agreement with previous measurements^5,6,8,9^. Using newly developed procedures, simulations are performed to support the interpretation of the experiments.

The approach used here employs a moderately powered (2 × 10^14^ W cm^−2^) 800 nm NIR pulse to ionize the parent molecule, such that molecular fragments produced in the UV photodissociation remain largely intact, and Coulomb curves associated with repulsion by the charged molecular or atomic partner can be readily observed. Combined with time-resolved covariance analysis to confirm photochemical channel assignments, this universal detection technique should be a valuable tool in exploring the photochemistry of more complex molecules in the gas phase. Questions still remain about the interpretation of the short time data, and the signal produced from NIR ionization of intact, but energized parent molecules. Understanding the short time dynamics may require stronger NIR ionization, leading to more complete atomization of the parent molecule. This approach is emerging as a valuable tool in probing the short-time structural changes in the excited parent molecule subsequent to UV photon absorption^24^. It seems reasonable to suppose that as molecular complexity increases, a greater range of experimental tools and techniques will be required to unravel their chemical dynamics.

## Methods

### Experimental

The experiments were conducted using a newly built time-resolved CEI apparatus. The optical set-up consisted of a Ti:Sapphire laser system (Spectra Physics Solstice Ace) with a central wavelength of 800 nm and a bandwidth of 35 nm. The laser beam was split and the dominant fraction was used to pump an optical parametric amplifier (Spectra Physics TOPAS Prime), set up here to generate the 240 nm (2.8 nm bandwidth) pump laser pulse, whilst the smaller fraction was used as the probe laser pulse. The pump pulse had an energy of 8 μJ and a peak intensity of 4 × 10^12^ W cm^−2^, and the probe pulse an energy of 35 μJ and a peak intensity of 2 × 10^14^ W cm^−2^. The peak intensity of each pulse was calculated based on its energy, duration and focal spot size, assuming a Gaussian beam profile. The pump pulse was focused with a 500 mm focal length lens and the probe pulse with a 300 mm focal length lens. The two pulses were overlapped through use of a dichroic mirror and then pass into the VMI spectrometer^25,26^.

The design of the spectrometer has been described in a previous publication^31^. A mixture of nitrobenzene diluted in helium was prepared by passing helium gas through liquid nitrobenzene inside a custom-built bubbler, based on the design of Shaikh et al.^32^. A supersonic molecular beam was produced by expanding the gaseous mixture into the vacuum chamber through a pulsed valve (Series 9 General Valve) and collimating the expansion with a skimmer. The molecular beam entered the VMI ion optics through an aperture in the rear of the repeller electrode and, in the region between the repeller and extractor electrodes, was intersected perpendicularly by copropagating pump and probe pulses.

The field generated by the ion optics accelerates the nascent ions onto a detector consisting of a pair of chevron-stacked microchannel plates coupled to a P47 phosphor screen, light from which was monitored by a pixel imaging mass spectrometry (PImMS) camera^33,34^ equipped with a PImMS2 sensor. The PImMS2 camera recorded both the two-dimensional (2D) position and time of each ion impact with a precision of 25 ns. This allowed images of all ions species to be recorded simultaneously, with the timing of each event indicative of the particle’s mass-to-charge ratio. The experiment was restricted to operate at 20 Hz due to the limited repetition rate of the PImMS2 camera.

The time delay between the two laser pulses was controlled by a motorized linear delay stage in the probe laser beam path. The position of temporal overlap was established in-situ based on the delay-dependent enhancement in the yield of H_2_O^+^ from non-resonant, two-color multiphoton ionization by the pump and probe pulses^35^. From the width of the cross-correlation peak an instrument response function of 97 ± 12 fs was determined. A frequency resolved optical gating (FROG)^36^ measurement of the probe pulse was used to determine a pulse duration of 42 ± 2 fs. Based on this measurement, together with the observed UV-NIR cross-correlation time, we estimate the UV pulse duration to be 95 ± 12 fs.

### Image processing

To produce the time-resolved momentum distributions seen in Fig. 1, the ion images at each delay step were Abel-inverted using the pBASEX algorithm^37^ to obtain a set of radial distributions. Each radial distribution was then normalized by the number of laser shots and finally converted from radius to momentum. The absolute kinetic energy scale of the velocity-map ion images was calibrated based on the spacing of the above-threshold ionization peaks in the measured photoelectron spectrum of argon^38^. Further details of the image processing methods employed are given in the Supplementary Note 2.

### Covariance mapping

In this work, we examine not only the momenta of the various fragment ions, but also the correlated momenta of pairs of fragment ions. This was achieved by calculating their covariance^22,39^—a statistical measure of the linear correlation between parameters across a data-set of many observations (laser shots). The two-fold covariance between a pair of variables is defined as:

4 $$\begin{array}{rcl}{{\rm{cov}}}(A,B)&=&\langle (A-\langle A\rangle )(B-\langle B\rangle )\rangle \\ &=&\langle AB\rangle -\langle A\rangle \langle B\rangle ,\hfill\end{array}$$

where 〈*i*〉 refers to the mean of the measured quantity *i* over a series of observations. The first term of Eq. (4), 〈*AB*〉, is the correlated product, calculated from all pairs of ion *A* and ion *B* detected in the same laser shot. If multiple molecules are probed per laser shot, the correlated product will contain contributions from ions which are falsely correlated because they originate from different parent molecules. This false correlation background is accurately captured by the uncorrelated product 〈*A*〉〈*B*〉, which can be calculated from all pairs of ion *A* and ion *B*, independent of whether they are detected in the same laser shot or not. By subtracting the uncorrelated product from the correlated product, the true ion correlations are isolated.

Calculating the covariance between the 2D projected momentum vectors of a pair of ions, as has been carried out in this work, yields a four-dimensional covariance map. Because this is difficult to visualize, the covariance map is often transformed into a representation with a reduced number of dimensions^22,40,41^. In Fig. 3, only the projected momentum of one of the ions from the pair is displayed.

Due to the nature of covariance analysis, it relies on stable experimental conditions. Fluctuations in any experimental parameter which is correlated with the total ion signal will cause the yield of all species to rise and fall as one, introducing false contributions to the calculated covariance. To account for this effect, we rely on contingent covariance analysis^42^. This involves grouping the raw data into smaller subsets over which the total ion count per laser shot is approximately constant^43^, calculating the covariance separately for each subset, and then averaging the resulting set of covariance maps to give the final result.

### Kinetic fitting

The kinetic traces shown in Fig. 4 where obtained by integrating the time-resolved momentum distributions over particular momentum ranges of interest, as highlighted in the top panels of the figure. The kinetic traces were then fit using appropriate equations to describe the decay or rise in signal as a function of time. Note that the time constant data shown in Table 1 were obtained by fitting two or three time-ranges simultaneous to yield a single global set of parameters. Further information about the methods used for the fitting and the kinetic equations employed are given in the Supplementary Note 5.

## Supplementary information

Supplementary material

## Data Availability

Data are made available through the Oxford Research Archive (ORA) (10.5287/ora-gj8yx7xb0).
