# Supplementary material for: The femtochemistry of nitrobenzene following excitation at 240 nm
Source: Commun Chem. 2025 Sep 1;8:268. doi: 10.1038/s42004-025-01672-2 (PMC12402474; doi:10.1038/s42004-025-01672-2)
Supplement: Supplementary file 1 — Supplementary material [file 42004_2025_1672_MOESM1_ESM.pdf]

Supplementary information: The femtochemistry of nitrobenzene following excitation at 240 nm

Chow-Shing Lam,[1] Tai-Che Chou,[1] Joseph McManus,[1] Ciara Hodgkinson,[1] Michael Burt,[1,2] and Mark Brouard[1*]

[1] *Department of Chemistry, University of Oxford, The Chemistry Research Laboratory, 12 Mansfield Road, Oxford OX1 3TA, United Kingdom*

[2] *Department of Chemistry, Trent University, 1600 West Bank Drive, Peterborough, K9L 0G2, ON, Canada*

[*] *Corresponding author. E-mail: mark.brouard@chem.ox.ac.uk*

Table of contents

S1 Time-of-flight spectra and ion images

S2 Image processing

S3 Charge assignment and momentum distributions

S4 Covariance maps

S5 Kinetic analysis

S6 Heuristic simulation for UV-induced channels

Supplementary Methods

S1 Supplementary Note 1: Time-of-flight spectra and ion images

Example time-of-flight (ToF) spectra recorded with the UV laser only, and for positive (UV-early) and negative (UV-late) time delays, are shown in Fig. S1. Charged species were identified through a calibration between ToF and mass-to-charge ratio (m/z). Our analysis focused on ion species corresponding to the three dissociation channels reported previously in the literature [1, 2], and identified in the main text. Example ion images are also shown in Fig. S1. As indicated in the figure, they were obtained by selecting data within a specific ToF (m/z) time-window within the PImMS data set.

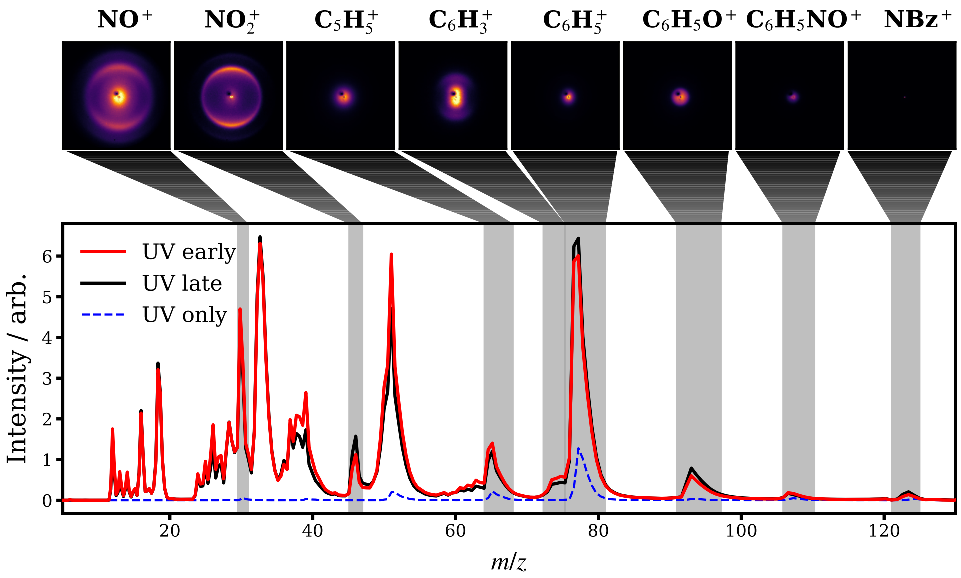

Figure S1: **Time-of-flight spectra and ion images.** The red, black, and blue dashed curves represent time-of-flight spectra under different conditions: red for positive pump-probe delay times, black for negative delay times, and blue for the UV pump pulse only. The peak laser intensities employed are given in the main text. Representative ion images obtained by integrating the PImMS data over the mass ranges indicated, are also shown above the ToF spectra.

The PImMS data set was further filtered based on the pump-probe delay time, yielding

time-resolved mass spectra. Fig. S2 illustrates such an analysis, in which the negative delay time data has been subtracted from the positive delay time data, thereby highlighting which mass peaks are enhanced when the UV laser pulse arrives before the NIR laser pulse (red signal), and which are depleted (blue signal).

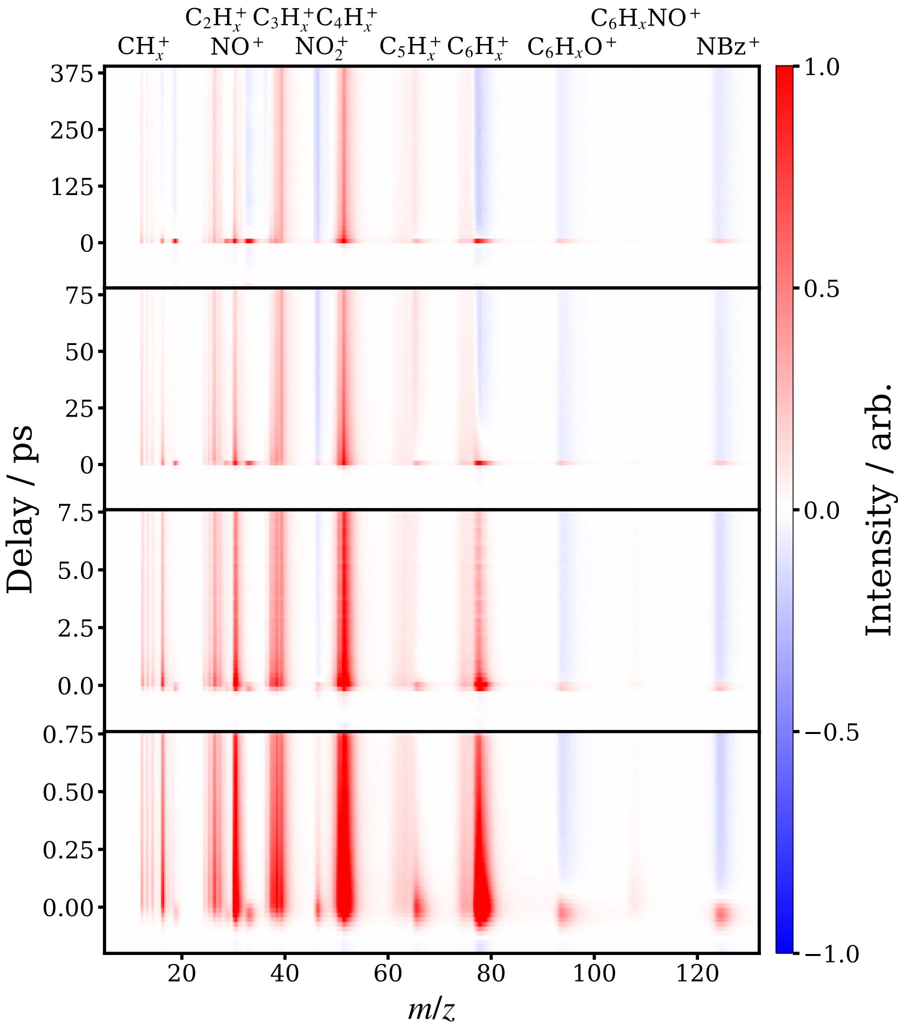

Figure S2: **Time-resolved mass spectra.** The panels show ToF spectra for pump-probe delay scan ranges of 500 ps (top), 100 ps, 10 ps, and 1 ps (bottom). Each ToF spectrum has been baseline-corrected by subtracting the average signal prior to time zero, highlighting signal enhancements (red) or depletions (blue) for different ion species.

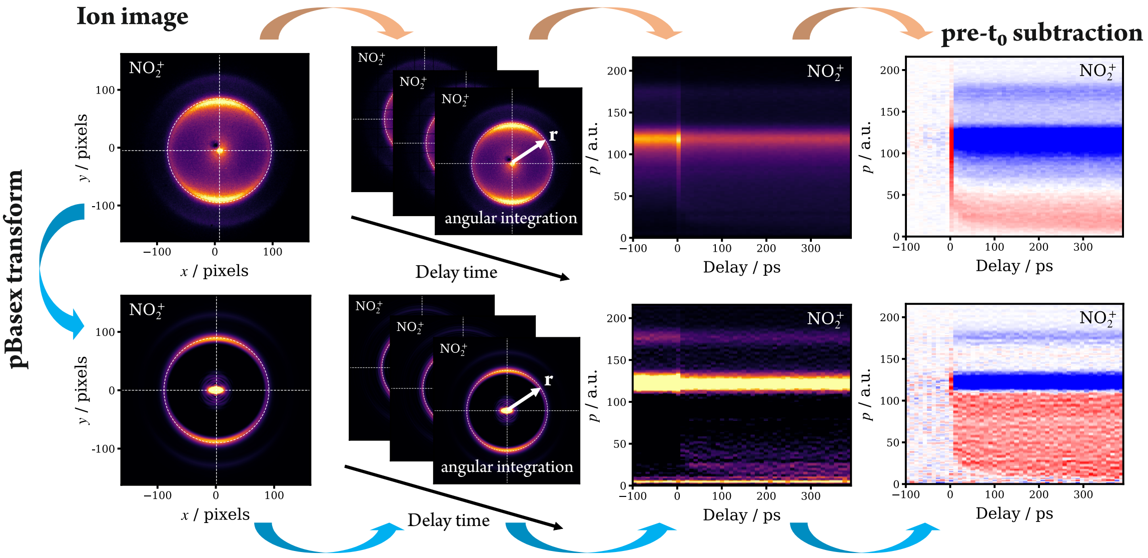

Figure S3: **Flowchart of the image processing procedure.** The top row illustrates the generation of time-resolved radial distributions, while the bottom row shows the corresponding momentum distributions obtained *via* Abel-inversion using pBasex [3].

S2 Supplementary Note 2: Image processing

The image analysis procedure is illustrated schematically in Fig. S3. To visualize the pump-probe delay time and momentum dependence of the ion yields, an ion image for the particular mass peak was generated at each pump-probe delay. The corresponding momentum distribution was obtained by integrating over all angles and then plotted as a function of delay time. This analysis was performed on both the raw ion images, yielding the time-dependent radial (or 'projected momentum') distributions [4] (see data shown in Fig. 3 of the main text), and on the pBasex-inverted [3] ion images, yielding the time-dependent momentum distributions (see Fig. 1 of the main text).

S3 Supplementary Note 3: Charge assignment and momentum distributions

The time-resolved momentum distributions exhibited three distinct components: features with a constant momentum as a function of increasing delay time, Coulomb curves, and ground state bleaching (GSB) channels. These were labeled I-IV, *A*, *B* and *C*, and GSB, respectively, in Fig. 1 of the main text.

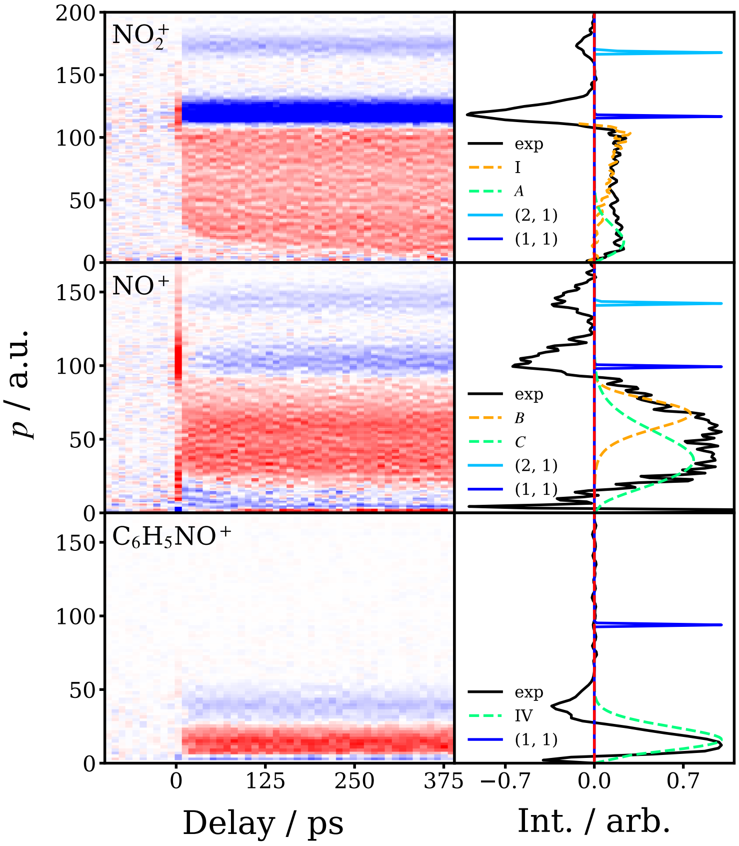

Figure S4: **Channel assignments for NO_2^+, NO^+ and $C_6H_5NO^+$.** The right column compares momentum distributions integrated over the last 100 ps (black lines) with simulated distributions for the GSB features corresponding to singly charged $(1, 1)$ and doubly charged $(2, 1)$ cofragments. The labels in the right hand panels refer to the various dissociation channels referred to in the main text (see Fig. 1). The dashed colored lines indicate the asymptotic momenta calculated based on the kinetic energy distributions reported by Lin *et al.* [2] (see Section S6).

To verify the origin of the GSB signals, a classical simulation was performed for the two-body breakup process (see Fig. S4). The Coulomb explosion dynamics were modelled

using classical trajectory simulations, treating the interacting fragments as point charges located at their respective centres-of-mass [5]. A total of 10,000 individual events were simulated. To account for the initial random orientation of the nitrobenzene molecule, each event incorporated a random rotation to reflect the arbitrary spatial configuration of the parent molecule.

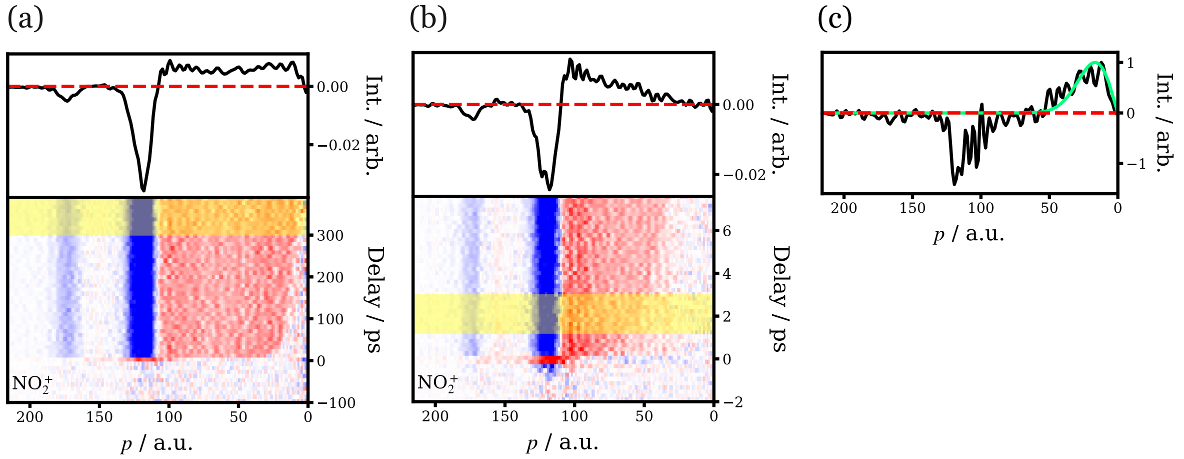

Figure S5: **Asymptotic momentum distribution for NO_2^+.** Momentum distributions integrated over delay times of (a) 300–400 ps and (b) 1.2–3.0 ps. (c) Difference between the momentum distributions in (a) and (b). The green line represents the asymptotic momentum distribution derived from the kinetic energy distribution reported by Lin *et al.* [2] following excitation at 248 nm.

The charge states of the simulated GSB features (the latter are shown in blue in Fig. S4) are denoted as (i, j), where i represents the charge of the cofragment and j the ion of interest (see right panels of Fig. S4). The GSB simulations for NO_2^+, NO^+, and $C_6H_5NO^+$ were based on the ground state equilibrium geometry of nitrobenzene [6]. To match the momentum distribution from the simulation to that from the experiment, we introduced a factor of the effective charge, 0.94 for the simulation of NO_2^+ (1, 1), 0.81 for NO^+ (1, 1) and 1.0 for $C_6H_5NO^+$. The simulated momenta for the GSB features closely matched the experimental data, with one exception: the simulated $(1, 1)$ channel for $C_6H_5NO^+$ did not align with the experimental GSB feature, consistent with the charge state of the GSB feature for this fragment being $(0, 1)$.

For the channels enhanced when the UV laser pulse precedes the NIR laser pulse (shown

in red in Fig. S4), momentum distributions of both the momentum-invariant channels and the Coulomb curves were determined in the asymptotic region using the last 100 ps of data in the 500 ps delay scans (see Section S6). The experimental asymptotic momenta showed good agreement with those determined using the kinetic energy distributions previously measured in the work of Lin *et al.* [2], as shown in the right panels of Fig. S4.

In Fig. 2 of the main text, the experimental asymptotic momentum of NO_2^+ was processed before comparison with previous results [2]. The momentum distribution integrated over the last 100 ps exhibits a bimodal structure (see Figs. S4 and S5), suggesting contributions from multiple channels. Furthermore, the higher momentum channel (labelled feature I in Fig. 1 of the main text) has a near constant intensity over the 500 ps of the current experiments (as will be discussed further in Section S5, in relation to the kinetic analysis to be shown in Figs. S10 and S11), whilst the lower momentum portion, associated with the NO_2^+ Coulomb curve increases in intensity over time, reflecting the generation of neutral NO_2 in the molecular photodissociation of nitrobenzene. To isolate this Coulomb curve from the time-invariant feature we subtracted the contribution of feature I from the final distribution, as illustrated further in Fig. S5. Since the momentum distribution of feature I remains unchanged throughout the pump-probe window, its distribution at 2 ps was used as a reference for subtraction. This approach ensures that only the relevant contributions are retained for direct comparison with the previous energy distributions reported by Lin *et al.* [2]. We assume that feature I is associated with a minor channel involving the NIR-induced Coulomb explosion of the *stable* parent ion generated by the UV pulse.

S4 Supplementary Note 4: Covariance maps

The time-dependent momentum distributions of the covariant fragment pairs ($C_6H_5^+$, NO_2^+), ($C_6H_5O^+$, NO^+), and ($C_5H_5^+$, NO^+) are shown in Fig. 3 of the main text. To further understand the momentum distributions of the ions, both recoil frame covariance maps [7]

and Newton plots of the covariances [8,9] are shown. Momentum filtering [10] was applied to both the recoil frame and the Newton plots of the covariances in order to isolate the signal of interest.

S4.1 Covariances with NO_2^+

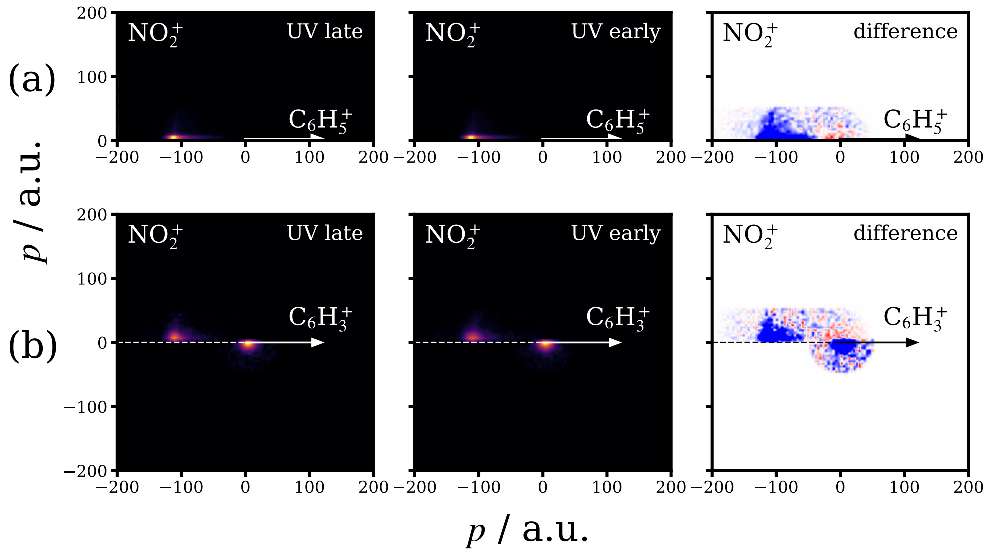

Figure S6: **Recoil frame and Newton plots for the covariant momentum distribution of (a) ($C_6H_5^+$, NO_2^+), (b) ($C_6H_3^+$, NO_2^+).** Note that for the covariance (X, Y) the momentum of Y is plotted with respect to X as the reference. The left column shows the covariance integrated over delay times before time-zero (UV late), the middle column represent the covariance integrated over delay times of 10–400 ps (UV early), and the right columns display the difference plot obtained by subtracting the left columns from the middle ones. For the difference plot, red and blue represent positive and negative covariance signals, respectively.

Fig. S6 shows the NO_2^+ covariances with $C_6H_5^+$ and $C_6H_3^+$. The ($C_6H_5^+$, NO_2^+) is seen to be back-to-back, as expected from momentum conservation, with the momentum distribution at positive delay times shifted towards lower momenta with respect to that observed at negative delay times (as shown also in Fig. 3 of the main text). Note that the signal at negative delay times is dominated by that from only the NIR probe laser. The momentum distribution for the ($C_6H_3^+$, NO_2^+) covariance map is broader, both in magnitude and angular range, than that for ($C_6H_5^+$, NO_2^+), reflecting the momentum carried away by the unobserved neutral H_2 or 2H species (see also Section S4.3).

S4.2 Covariances with NO^+

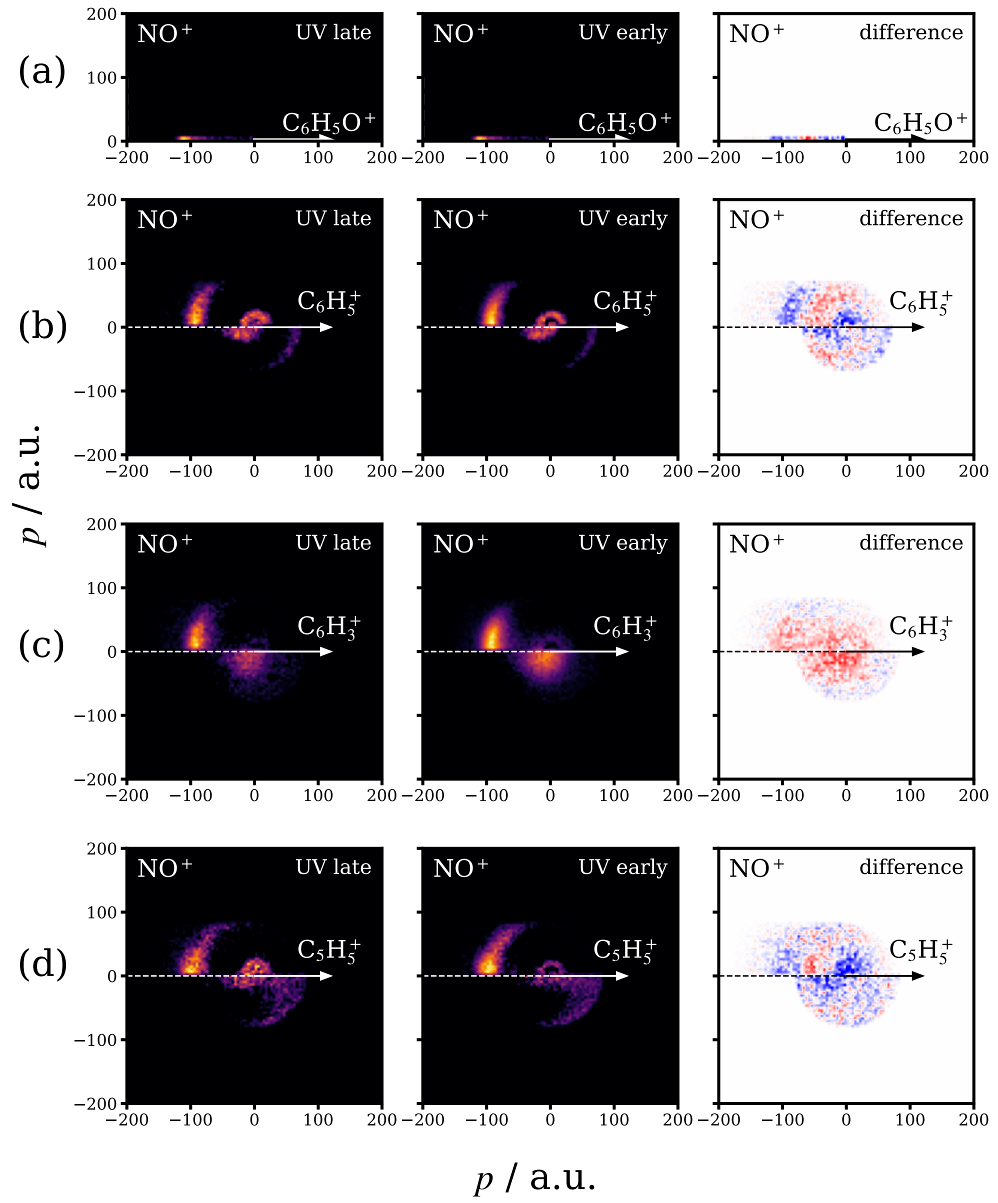

Figure S7: **Recoil frame and Newton plots for the covariant momentum distribution of (a) ($C_6H_5O^+$, NO^+), (b) ($C_6H_5^+$, NO^+), (c) ($C_6H_3^+$, NO^+), (d) ($C_5H_5^+$, NO^+).** Note that for the covariance (X, Y) the momentum of Y is plotted with respect to X as the reference. The left column shows the covariance integrated over delay times before time-zero (UV late), the middle column represents the covariance integrated over delay times of 10–400 ps (UV early), and the right column displays the difference plots obtained by subtracting the data in the left column from that in the middle. For the difference plots, red and blue represent positive and negative signals, respectively.

Covariances with NO^+ are shown in Fig. S7. As expected, the ($C_6H_5O^+$, NO^+) covariances are seen to be back-to-back and are shifted towards lower momentum compared to the

probe-only data, consistent with the data shown in Fig. 3 of the main text. The covariances for the fragment pairs ($C_6H_5^+$, NO^+) and ($C_6H_3^+$, NO^+) show progressive broadening of the momentum distributions consistent with the momentum taken away by the neutral third body, O atoms or O atoms and H_2/2H. The arcing feature clearly visible in the ($C_6H_5^+$, NO^+) Newton plot of the covariance is consistent with a time delay of the order of a rotational period between generation of $C_6H_5O^+$ and loss of O to generate $C_6H_5^+$ [9,10]. Finally, the ($C_5H_5^+$, NO^+) covariance is quite tightly confined to the back-to-back region, consistent with relatively prompt dissociation of *neutral*, internally excited C_6H_5O by loss of CO to form C_5H_5 (see following subsection).

S4.3 Time-resolved covariances

Time-resolved covariant momentum distributions are shown in Fig. S8. The data should be compared with those shown in Fig. 3 of the main text. The data further illustrate how loss of neutral species from the ions generated by the NIR pulse leads to a broadening of the covariant (projected) momentum distributions.

The broadening of the momentum distributions by loss of neutral species is further illustrated for NO_2 and NO in Fig. S9, which shows the projected momentum distributions obtained from the raw ion images and from the covariance data for a number of ions observed in the experiment. The distributions shown are obtained from data integrated over the final 300 ps of delay times from the 500 ps scans. In the case of NO_2^+, shown in panel (a) of Fig.S9, note that the projected momentum distribution obtained from the ($C_6H_5^+$, NO_2^+) covariance is significantly sharper than that observed in ($C_6H_3^+$, NO_2^+) covariance, reflecting the momentum imparted to the neutral species 2H/H_2 when $C_6H_5^+$ decays to $C_6H_3^+$.

The NO^+ covariance data (panel (b) of Fig. S9) show more complex behaviour, but generally reveal broadening momentum distributions with increasing loss of neutral species, either O or H_2/2H from the $C_6H_5O^+$ ion formed by the NIR pulse, or from the neutral,

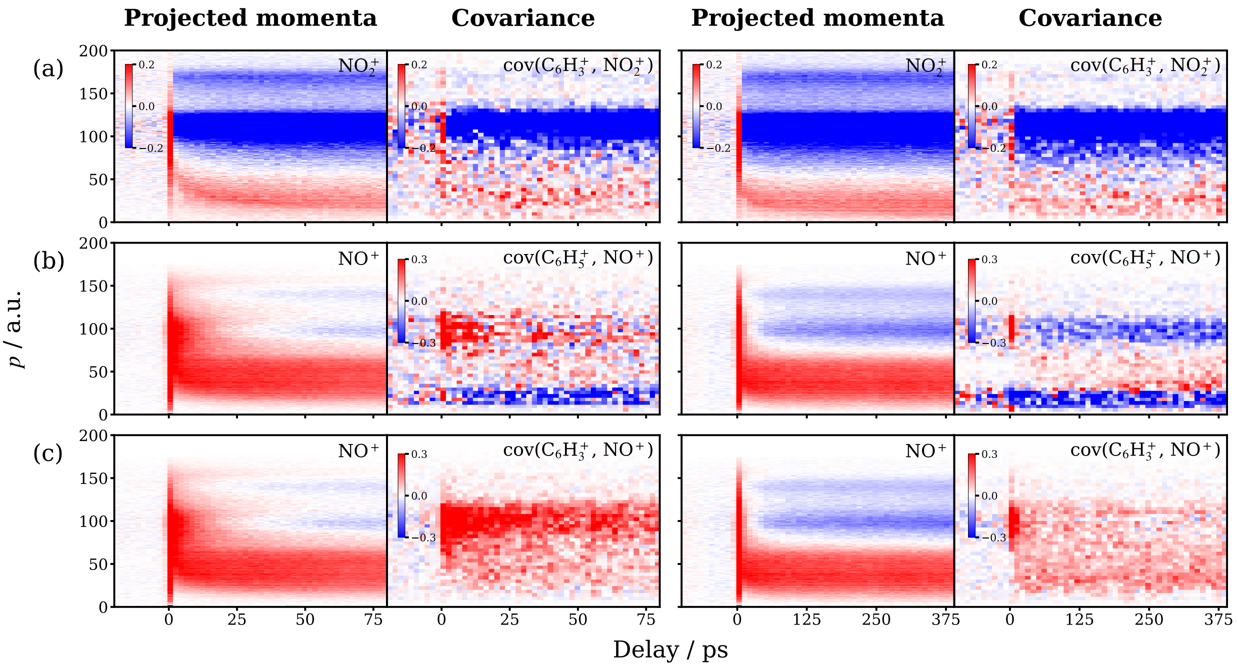

Figure S8: **Comparison between the time-resolved radial distributions (without Abel inversion) and the time-dependent covariances over the time-ranges of 100 ps (left panels) and 500 ps (right panels).** (a) Projected momentum of NO_2^+ and covariance ($C_6H_3^+$, NO_2^+). (b) Projected momentum of NO^+ and covariance ($C_6H_5^+$, NO^+). (c) Projected momentum of NO^+ and covariance ($C_6H_3^+$, NO^+).

internally excited (translationally cold) C_6H_5O products of the UV photodissociation of nitrobenzene, which go on to dissociate into C_5H_5 + CO (Channel 2(b) of the main text). The fact that the momentum distribution derived from the ($C_5H_5^+$, CO^+) covariance shows both fast, delayed and slow, prompt components indicates that these products (arising from Channel 2(b) of the main text) cannot be assigned exclusively to either one of these components. Therefore, the simplest explanation for these observed momentum distributions is that C_6H_5O + NO fragments from channel 2(a) are produced *via* both the fast 'triplet' and slow 'roaming' mechanisms, leading to the fast and slow momentum features B and C, and that the more internally energized C_6H_5O fragments arising from either mechanism preferentially go on to dissociate to C_5H_5 + CO. Coulomb explosion simulations of the Newton plots support the general picture outlined above. It should also be noted that three-fold covariance data would be valuable in assigning Channels 2(a) and (b) to specific momentum ranges, but under the current experimental conditions we were unable to record three-fold

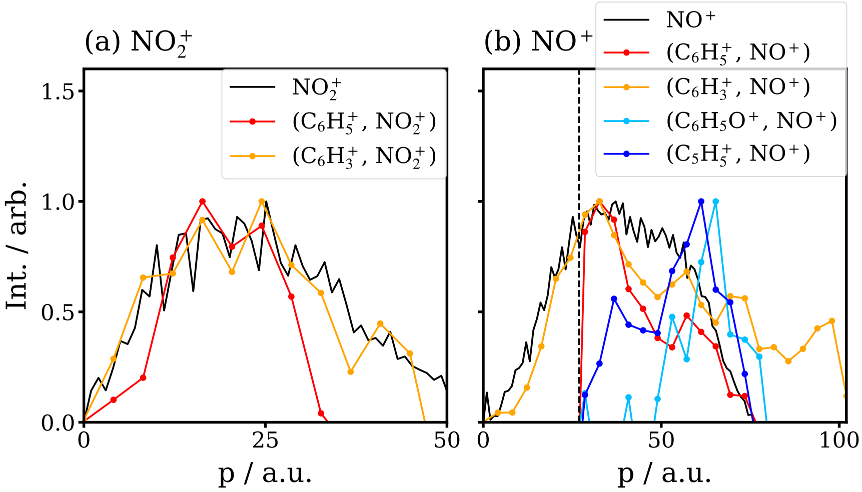

Figure S9: **Overlay of the projected momentum distributions (without Abel inversion) and the momentum distributions of covariances integrated from the pump-probe delay time of 100 ps to 400 ps.** Panel (a): The projected momentum distribution for NO_2^+ (black), and the projected covariant momentum distribution for ($C_6H_5^+$, NO_2^+) (red), and ($C_6H_3^+$, NO_2^+) (orange). Panel (b): The projected momentum distribution for NO^+ (black), and the projected covariant momentum distribution for ($C_6H_5^+$, NO^+) (red), ($C_6H_3^+$, NO^+) (orange), ($C_6H_5O^+$, NO^+) (light blue), and ($C_5H_5^+$, NO^+) (dark blue). The black dashed line indicates a cut-off in the experimental data to low momenta arising from the presence of a strong GSB signal.

covariances with sufficient quality to address this question.

S5 Supplementary Note 5: Kinetic analysis

To quantify the kinetics of the channels involved, we applied a global fitting function to fit simultaneously the data sets from multiple pump-probe time delay ranges. The data sets were fit using a damped least-squares fit procedure to a combined function of exponential rises (r) or decays (d), $f_i(t)$, as expressed below:

$$f_i(t) = c_i \times \left[\sum_{j=0}^{n} I_{d,j}(t) + \sum_{k=0}^{m} I_{r,k}(t) \right], \tag{1}$$

where c_i is a scaling factor of the combined function for each pump-probe time scale, i. $I_{r,k}(t)$ and $I_{d,j}(t)$ are the time evolutions of an ion product described by first-order rise and decay kinetics, respectively, which were convoluted with our instrumental response function,

assumed to be a Gaussian function [11]:

$$I_{d,j}(t) = \frac{A_j}{2} \times \exp\left(-\frac{(t-t_0)}{\tau_j} + \frac{\sigma^2}{2{\tau_j}^2}\right) \times \left[1 + \mathrm{erf}\left(\frac{t-t_0}{\sqrt{2}\sigma} - \frac{\sigma}{\sqrt{2}\tau_j}\right)\right] \tag{2}$$

$$\begin{aligned} I_{r,k}(t) &= \frac{A_k}{2} \times \left\{1 + \mathrm{erf}\left(\frac{t-t_0}{\sqrt{2}\sigma}\right)\right. \\ &\left. - \exp\left(-\frac{(t-t_0)}{\tau_k} + \frac{\sigma^2}{2{\tau_k}^2}\right) \times \left[1 + \mathrm{erf}\left(\frac{t-t_0}{\sqrt{2}\sigma} - \frac{\sigma}{\sqrt{2}\tau_k}\right)\right]\right\}. \end{aligned} \tag{3}$$

Here A_i is the pre-exponential factor and τ_i is the lifetime for process i, σ is the standard deviation of the Gaussian function (38 fs) used to fit the cross-correlation signal of the delay-dependent enhancement in the yield of H_2O^+, t_0 is the initiation time of the exponential rise or decay, and erf is an error function.

Table S1: **Summary table of the kinetic rise times.** Summary table of the kinetic rise times associated with the chemical channels identified (also see main text Fig. 1 for the assignments). All time constants are in picoseconds, and error limits represent 2σ. A selection of the kinetic data and fits are shown in Figs. S10 – S15, and in Fig. 4 of the main text.

Ion	Channel	τ_1 (A_1%)	τ_2 (A_2%)	τ_3 (A_3%)
NO_2^+	*A*	0.25 ± 0.05 (57 ± 3)	8.1 ± 1.2 (43 ± 4)	> 2000 (n.a.)
NO^+	*B* (fast)	0.87 ± 0.10 (69 ± 3)	14.2 ± 2.4 (31 ± 4)	
	C (slow)	0.14 ± 0.02 (67 ± 3)	7.9 ± 0.4 (33 ± 1)	
$C_6H_5NO^+$	IV	0.42 ± 0.06 (43 ± 1)	16.5 ± 1.4 (57 ± 4)	

The returned time constant data are shown in Tables S1 – S3, which contain results for the chemical channel rise times, the decay times of a selection of the momentum invariant features, and the rise times associated with GSB signals, respectively. The associated data and fits are shown in Figs. S10 – S15. In these figures, the kinetic traces in the bottom-row panels represent integrated intensities from the momentum regions highlighted in yellow in the upper-row panels. Black circles indicate the mean values of the integrated intensity, while the red line represents the fitted curve. The red shaded area denotes the experimental uncertainty (2σ).

Table S2: **Summary table of the kinetic decay times.** Summary table of the kinetic decay times associated with the momentum invariant features labelled I and II (see main text Fig. 1). All time constants are in picoseconds, and error limits represent 2σ. A selection of the kinetic data and fits are shown in Figs. S10 – S15, and in Fig. 4 of the main text.

Ion	Channel	τ_1 (A_1%)	τ_2 (A_2%)
NO_2^+	I	1.5 ± 1.2 (33 ± 13)	> 10000 (67 ± 15)
NO^+	II*	0.35 ± 0.01 (63 ± 1)	11.8 ± 0.6 (26 ± 1)

*Channel II and GSB of NO^+ were fitted simultaneously.

The intensities associated with the various time- and momentum-dependent ion yields invariably show biexponential or triexponential kinetic behaviour. This is clearly evident from the data shown in Fig. 4 of the main text and here in in Figs. S10 – S15. Because the data for several time-windows were fit simultaneously, the multiple time constants necessary to reproduce the signal rises can be recovered with reasonable confidence. For many momentum features we observe a fast component, τ_1, for which there are several possible explanations. Some enhancement in ion yield is expected at short times, reflecting the increased ionization efficiency of the parent molecule after UV excitation. Whether this short risetime signal is associated with a Coulomb curve of interest or is associated with an overlapping signal arising from an enhancement in the observed ion is hard to distinguish in the present experimental data. However, with improved statistics compared with the data recorded here, it should be possible to perform the kinetic analysis directly on the covariance data, rather than the individual ion yields, and that would be useful in identifying the origin of some of these shorter, perhaps overlapping fast components.

Figs. S10 and S11 show the fits to the NO_2^+ momentum distributions associated with the Coulomb curve, labelled feature A in Fig. 1 of the main text, and the time-invariant momentum component, labelled feature I in Fig. 1. For feature A, note that the NO_2^+ intensity in the momentum range indicated continues to rise on the 500 ps timescale (right panel of Fig. S10), consistent with the discussion in Section 2.3 of the main text and the nanosecond risetime previously measure by Lin *et al.* [2]. In contrast, the higher momen-

Table S3: **Summary table of the GSB lifetimes.** Summary table of the lifetimes associated with the GSB features (see main text Fig. 1). All time constants are in picoseconds, and error limits represent 2σ. A selection of the kinetic data and fits are shown in Figs. S10 – S15.

Ion	Channel	τ_1 (A_1%)[a]	τ_2 (A_2%)[b]	τ_3 (A_3%)[b]
NO_2^+	GSB			11 ± 2 (n.a.)
NO^+	GSB[c]			260 ± 80 (11 ± 3)
$C_6H_5NO^+$	GSB	0.14 ± 0.03 (43 ± 4)	0.32 ± 0.22 (12 ± 2)	20 ± 2 (45 ± 6)

[a]Kinetic decay
[b]Kinetic rise
[c]Channel II and GSB of NO^+ were fitted together.

tum NO_2^+ feature shown in Fig. S11 is constant in intensity on the same timescale. These observations are consistent with the discussion of Section S3 concerning the deconvolution of the multi-component NO_2^+ momentum distributions shown in Fig. S4 and S5.

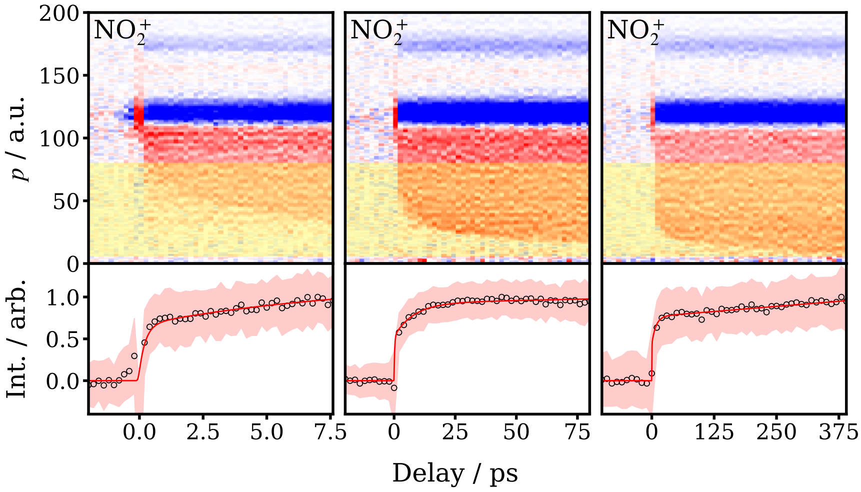

Figure S10: **Kinetic fitting of NO_2^+** over the momentum range of 6–80 a.u. highlighted with yellow shading in the upper panels. The fitted data are associated with feature *A*. The returned time constants shown in Table S1.

Exponential rises and decays were observed in the signals associated with the GSB features (see Table S3), where the former reduces ion intensity and the latter increases it. This behaviour can be explained qualitatively using a simplified kinetic model. The ion yield in the GSB feature originates from both the ground state and the initially populated excited state, with the latter exhibiting a higher ionization cross-section. When the pump laser

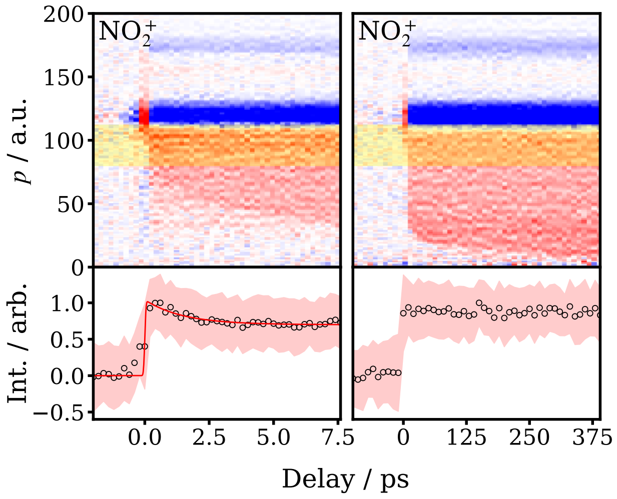

Figure S11: **Kinetic fitting of NO$_2^+$** over the momentum range of 80–112 a.u. highlighted with yellow shading in the upper panels. The fitted data are associated with feature I. The returned time constants shown in Table S2.

excites the ground-state population, the ion yield of the GSB feature increases within the pulse duration. Over time, the decay of the excited-state population reduces the ion intensity, eventually dropping below the UV late-region intensity level (where the pump-probe delay time is negative). Consequently, the kinetic rises and decays in Table S3 reflect the dynamics of intact nitrobenzene. However, due to the overlap in momentum distributions, it was not possible to isolate ion signals from different excited states (*e.g.*, S_4, S_1, T_3, and T_1). As a result, the lifetimes presented in Table S3 remain subject to further interpretation.

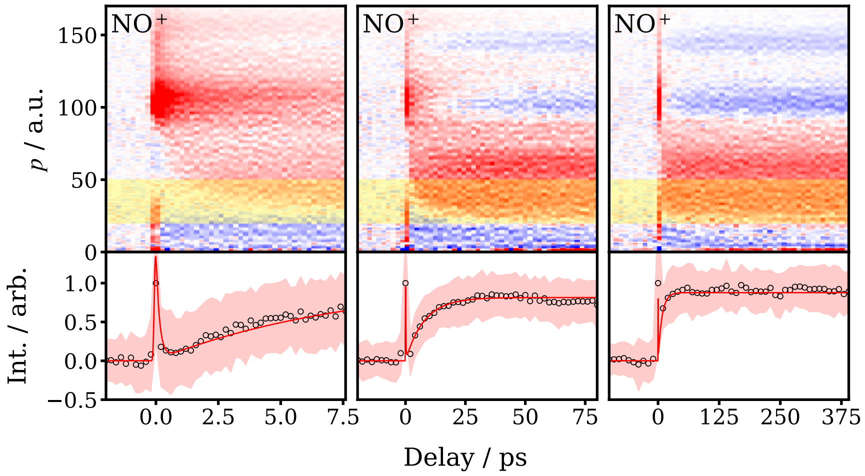

Figure S12: **Kinetic fitting of NO$^+$** over the momentum range of 20–50 a.u. highlighted with yellow shading in the upper panels. The fitted data are associated with feature C. The returned time constants shown in Table S1.

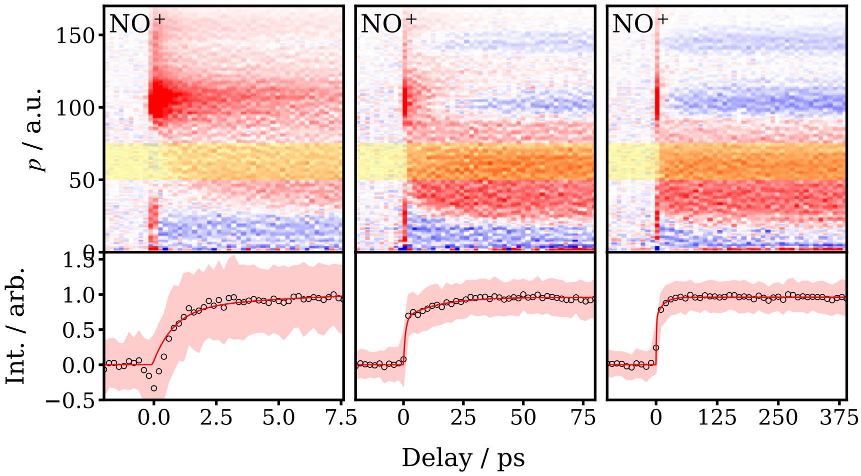

Figure S13: **Kinetic fitting of NO^+** over the momentum range of 50–75 a.u. highlighted with yellow shading in the upper panels. The fitted data are associated with feature B. The returned time constants shown in Table S1.

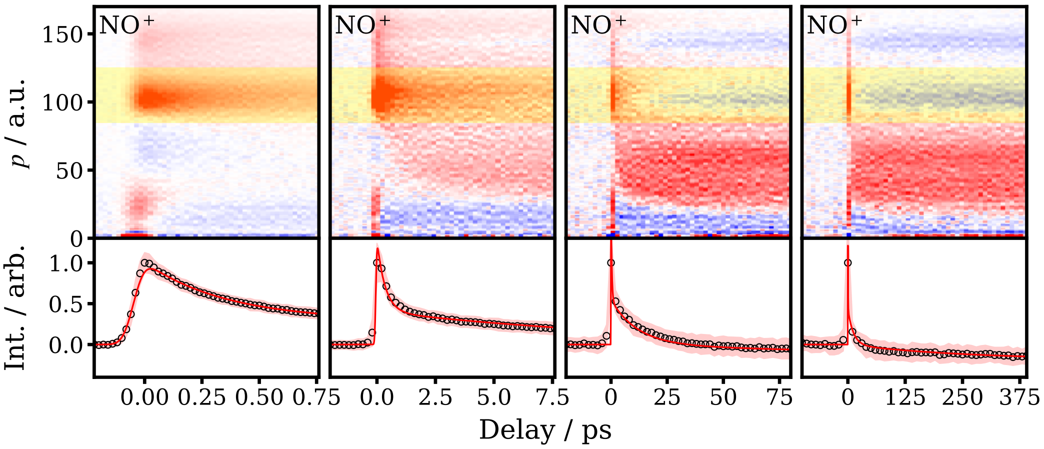

Figure S14: **Kinetic fitting of NO^+** over the momentum range of 85–125 a.u. highlighted with yellow shading in the upper panels. The fitted data are associated with feature II. The returned time constants shown in Table S2.

S6 Supplementary Note 6: Heuristic simulation for UV-induced channels

The Coulomb curves and momentum-invariant features observed in the time-resolved momentum distributions were modelled using a heuristic simulation. This simulation incorporated (a) the asymptotic kinetic energy distribution reported by Lin *et al.* [2] and (b) the time constants obtained from the kinetic analysis (Section S5). The simulation procedures for NO_2^+, NO^+, and $C_6H_5NO^+$ are detailed below, following a description of the general

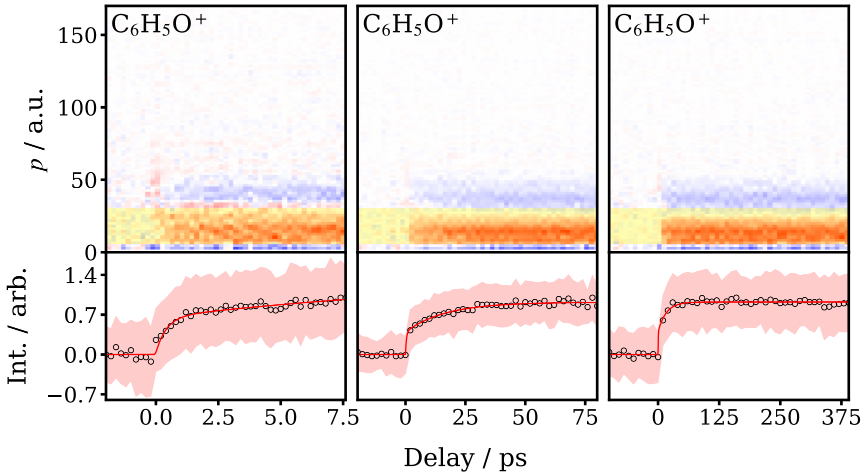

Figure S15: **Kinetic fitting of $C_6H_5NO^+$** over the momentum range of 6-30 a.u. highlighted with yellow shading in the upper panels. The fitted data are associated with feature IV. The returned time constants shown in Table S2.

procedures employed.

Note the simulations neglect a few features which are not pertinent to the main discussion. For both NO and NO_2, we have not included simulations of the signals associated with the enhancement and bleaching of the (2,1) ion channels at momenta above 125 a.u. These signals play little role in the interpretation of the data of interest, such as the Coulomb curves associated with the (1,1) channels. Nor have we included the bleaching of the low-momentum NO_2 and NO features at momenta below 25 a.u. – these signals arise as a result of the bleaching of the parent molecule by the UV pump laser, and again do not contain dynamical information of significance to the discussion. We have also neglected a detailed description of the early-time behaviour of ≤ 1 ps, focusing more on the description of the signals associated with the photochemical Channels 1-3, including Coulomb curves A, B and C, and feature IV.

S6.1 General simulation procedure

Fig. S16 illustrates the method used here for the heuristic simulation of the time-resolved momentum distributions. The figure takes as a specific example the modelling of the Coulomb curve for photodissociation of nitrobenzene yielding $C_6H_5 + NO_2$ (Channel (1) of the main

text).

The momentum (p) and time delay (t) dependent intensity distributions, $I(p,t)$, for specific rise features in the time resolved momentum data, such as those shown in Fig. 1 of the main text, are simulated using the expression:

$$I(p,t) = \int_0^{p_\infty^{\max}} \int_0^{t_\mathrm{B}=t} \rho\left(p_\infty\right) \cdot f'\left(t_\mathrm{B}\right) \cdot \delta\left(p - C\left(t : p_\infty, t_\mathrm{B}\right)\right) \,\mathrm{d}t_\mathrm{B}\,\mathrm{d}p_\infty\,, \tag{4}$$

where $\delta(\cdots)$ is the Dirac delta function, p_∞ is the asymptotic momentum and t_B is the time of birth of the fragment of interest. Here $I(p,t)$ is expressed as a double integral over a product of three terms, $\rho(p_\infty)$, $f'(t_\mathrm{B})$ and $C(p_\infty, t)$. $\rho(p_\infty)$ is the asymptotic momentum distribution of the ion of interest. We assume that this asymptotic momentum distribution, obtained at infinite time, corresponds to that generated in the neutral photodissociation process, which in this work is taken from the literature, as discussed further in the subsections below. Furthermore, we assume that the neutral momentum distribution does not vary with time delay. $f'(t_\mathrm{B})$ is the derivative of the kinetics with respect to t_B for the specific features in question. It represents the amount of signal of interest that is generated at time of birth, t_B, which in the case in question is the origin of the Coulomb curve associated with NO_2 formation at that particular time. In terms of the kinetic equations, $f(t_\mathrm{B})$ can be written approximated by:

$$f\left(t_\mathrm{B}\right) = \sum_{i=1}^{n} A_i^r \left[1 - \exp\left(\frac{-t_\mathrm{B}}{\tau_i}\right)\right] + \sum_{j=1}^{m} A_j^d \left[\exp\left(\frac{-t_\mathrm{B}}{\tau_j}\right)\right]\,. \tag{5}$$

In this expression, A_i are the weights of the rise or decay features, i or j, with time constants τ_i or τ_j. Note that the simulations employ slightly simplified kinetic equations to those employed in the kinetic fitting, in that they do not allow for the convolution with the Gaussian characterizing the laser cross-correlation, which was found unnecessary when simulating the 10 ps, 100 ps and 500 ps data.

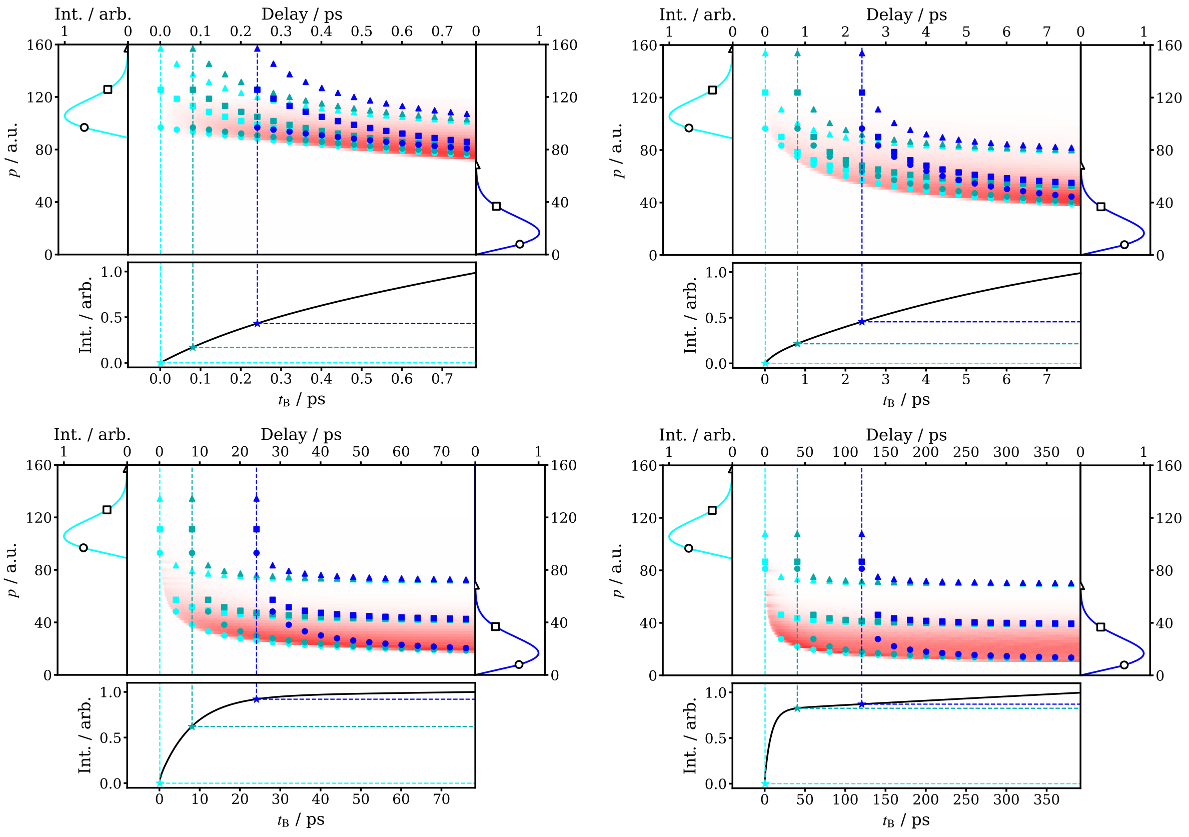

Figure S16: **Heuristic simulation intensity distributions associated with the Coulomb curve for the UV photodissociation yielding C_6H_5 + NO_2 across four time scales.** In each subplot, both the left and right panels depict the asymptotic momentum of NO_2. However, the left panel is offset by the momentum gained from the Coulomb explosion at $t = 0$, assuming a particular value of the initial charge separation, R. The middle panel presents the simulated Coulomb curve calculated using Eq. (4) (shaded red features), while the bottom panel shows the intensity integrated over all momentum ranges as a function of time of birth. Three asymptotic momenta, p_∞ and three times of birth, t_B were sampled for visualization and are shown as points in the middle panels of each subplot. See text for further details.

The final term in Eq. (4), $\delta\left(p - C\left(t : p_\infty, t_B\right)\right)$, defines the momentum, p, associated with the Coulomb curve. The momentum from the Coulomb curve itself is given by the equation:

$$C\left(t : p_\infty, t_B\right) = p_\infty + \sqrt{\frac{s \cdot 2\mu k e^2}{R + p_\infty\left(t - t_B\right)/\mu}}, \tag{6}$$

in which the first term accounts for the momentum gained from the UV photodissociation of nitrobenzene, and the second term corresponds to the momentum contribution from the Coulomb explosion. Here s represents the fraction of Coulomb explosion energy converted

into translational motion (defined in the following subsections), μ is the reduced mass, k is the Coulomb constant ($8.99 \times 10^9\,\mathrm{N\,m^2\,C^{-2}}$), e is the elementary charge, and R is the initial separation between the fragment charges, which in this work we locate on the center-of-masses of the fragments.

S6.2 Simulation for NO_2^+

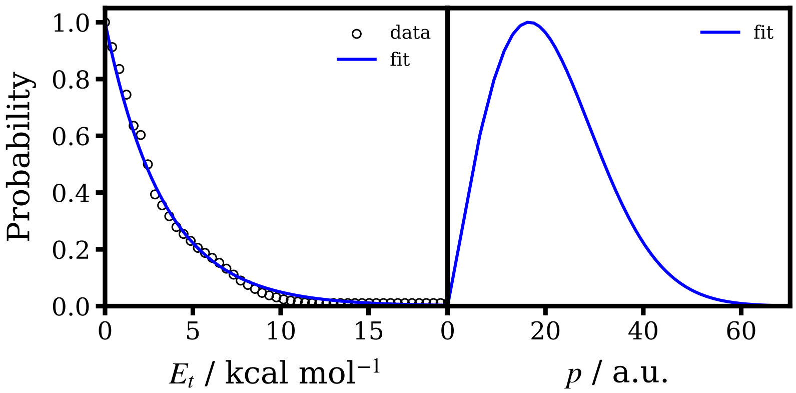

Figure S17: **Asymptotic distribution of NO_2^+.** The left panel presents the of the total kinetic energy release distribution, E_t. Experimental data (black circles) are adapted from Ref. [2], while the fitted Boltzmann distribution is shown in blue. The right panel depicts the corresponding function transformed into momentum space.

The first step in the simulation requires determining the asymptotic momentum distribution of the ion of interest, $\rho(p_\infty)$, in this case NO_2^+. In this work this was obtained by fitting a Boltzmann distribution to the asymptotic kinetic energy release reported by Lin *et al.* [2], as shown in Fig. S17. The fitted function was then converted from energy space to momentum space using the appropriate Jacobian.

To use Eq. (4) to simulate the Coulomb curve associated with the C_6H_5 + NO_2 channel, the value of R in Eq. (6) was set to 3.5 Å, based on the transition state structure on the potential energy surface of the triplet $^3(n_\mathrm{B}, \pi*)$ state [12], while s was set to 0.5 to match curvature of the experimentally observed Coulomb curve. It should be noted that although a value of $s = 0.5$ might indicate a significant fraction of the energy associated with the Coulomb explosion is channelled into internal degrees of freedom, the precise value used

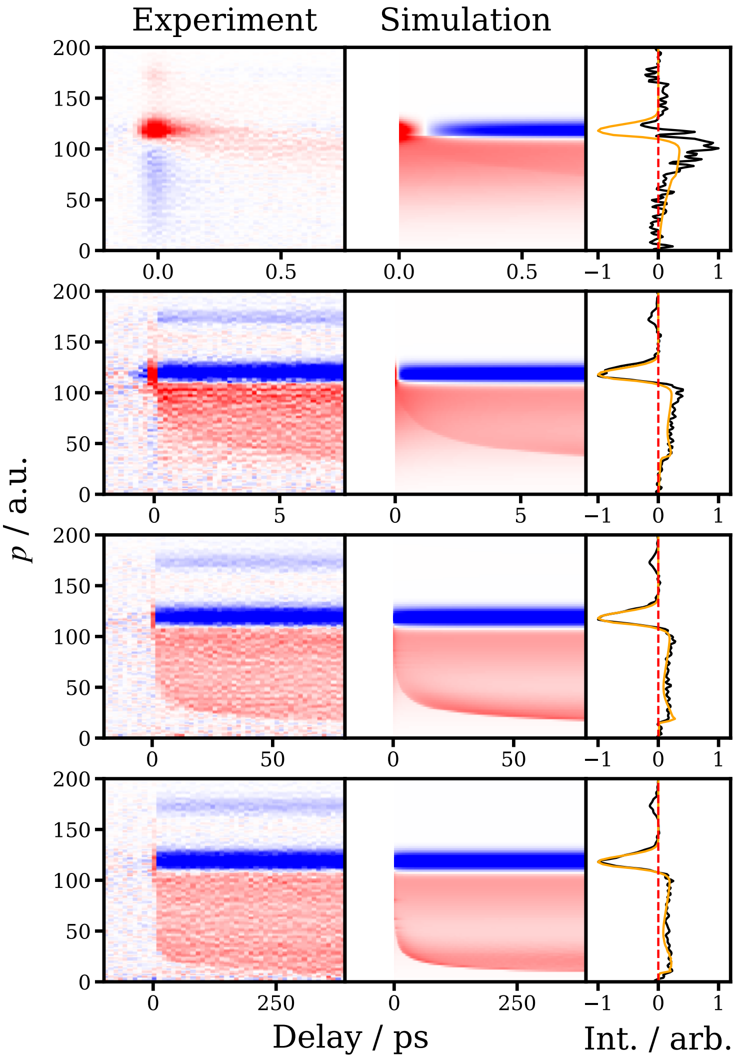

Figure S18: **Comparison of the time-resolved momentum distribution for NO_2^+ with the heuristic simulation incorporating channels A, I, and GSB.** In the left and middle columns, red and blue represent positive and negative signals, respectively. The right column displays the momentum distribution integrated over the final 10% of the pump-probe delay time, where the black line corresponds to experimental data and the orange line to the simulation.

in the simulation will also depend on assumptions made about the location of the charges on the parent molecule at the instant of Coulomb explosion. In this work, the simulations assume that the charges are initially located on the center-of-masses of the fragmenting ion pairs. Assuming a larger initial charge separation would require use of a larger value of s than that used here to reproduce the experimentally observed momentum distributions.

The momentum-invariant channel, labelled Channel I, and the GSB feature can also be

incorporated into the simulation using a reduced form of Eq. (4):

$$I(p,t) = \rho\,(p_\infty) \int_0^{t_\mathrm{B}=t} f'\,(t_\mathrm{B})\;\mathrm{d}t_\mathrm{B} = \rho(p_\infty) f(t) \equiv \rho(p) f(t)\,, \tag{7}$$

where $\rho(p)$ in Eq. (7) was obtained by fitting a Gaussian function to the momentum distribution of both channel I and GSB. Fig. S18 presents the complete simulation, including channel A (Coulomb curve), channel I, and the GSB feature. $f(t)$ is simply the kinetic equation associated with the momentum-invariant channel in question.

S6.3 Simulation for NO^+

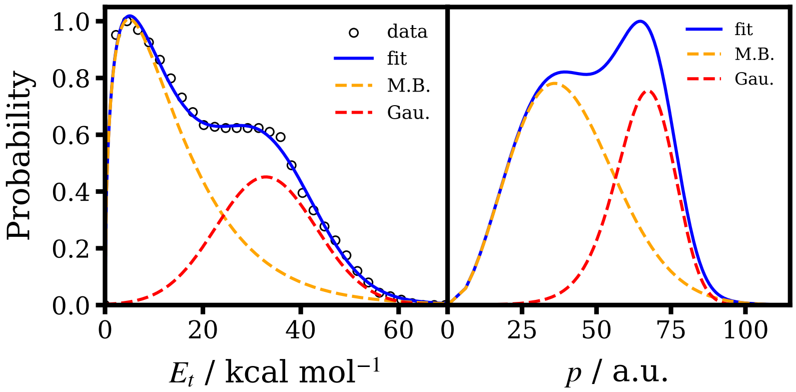

Figure S19: **Asymptotic momentum distribution for NO^+.** The left panel shows the probability distribution of the total kinetic energy release. Experimental data (black circles) are adapted from Ref. [2]. The fitted curve (blue line) consists of a sum of a Boltzmann distribution (M.B. - orange dashed line) and a Gaussian function (Gau. - red dashed line). The right hand panel presents the corresponding distributions in momentum space. See text for further details.

Similar to the procedure for NO_2, the asymptotic NO momentum distribution was derived from the total kinetic energy distribution presented in from Ref. [2]. As shown in Fig. S19, the energy distribution is bimodal and was fit by combining the Maxwell-Boltzmann distribution with a Gaussian function:

$$\rho(E) = A_1 \sqrt{\frac{4E_t}{\pi k_B^3 T^3}} \times \exp\left(-\frac{E_t}{k_B T}\right) + A_2 \times \exp\left(-\frac{(E_t - E_0)^2}{2\sigma^2}\right)\,, \tag{8}$$

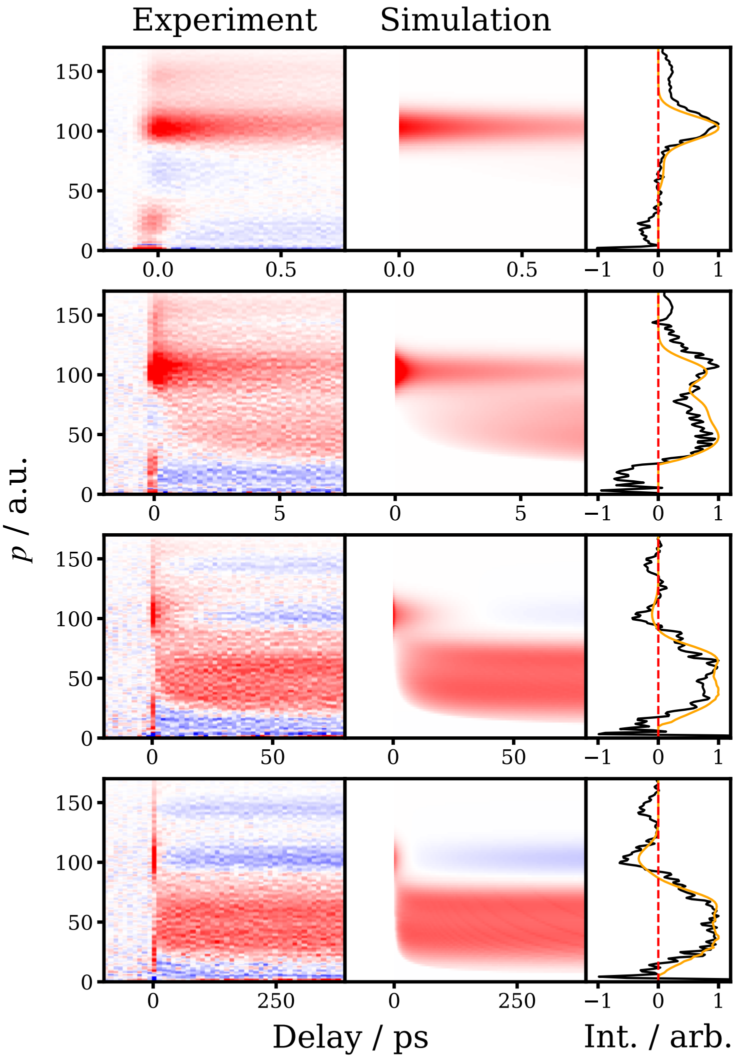

Figure S20: **Comparison of the time-resolved momentum distribution NO^+ with the heuristic simulation incorporating channels B, C, II, and GSB.** In the left and middle columns, red and blue represent positive and negative signals, respectively. The right column shows the momentum distribution integrated over the final 10% of the pump-probe delay time, where the black line corresponds to the experimental data and the orange line to the simulation.

where E_t is the total kinetic energy release, A_1 and A_2 are the magnitude of the Maxwell-Boltzmann function and the Gaussian function, respectively, k_B is the Boltzmann constant, T is the temperature, and E_0 and σ are the mean energy and width of a Gaussian distribution, respectively. This distribution was then transformed into momentum space.

The Coulomb curve features, calculated using Eq. (4), and the momentum-invariant features, calculated using Eq. (7), were then combined to generate the simulation shown in Fig. S20. The calculations employed a critical charge separation R of 3.33 Å based on the

transition state structure of the electronic ground-state nitrite isomer responsible for the roaming mechanism [12, 13]. The value of s was set to 0.3. Note that the translationally colder component of the momentum distribution exhibits a faster rise time (7.9 ± 0.4 ps) than the translationally hotter component, which shows a slower rise time (14.2 ± 2.4 ps). The resulting simulations are shown in Fig. S20, in which they are compared with the experimentally derived time-resolved momentum data.

S6.4 Simulation for $C_6H_5NO^+$

Similar to the process used for the other chemical channels, the asymptotic momentum distribution was obtained by fitting the total kinetic energy distribution from Ref. [2] to a Maxwell-Boltzmann distribution and then transforming to momentum space (see Fig. S21).

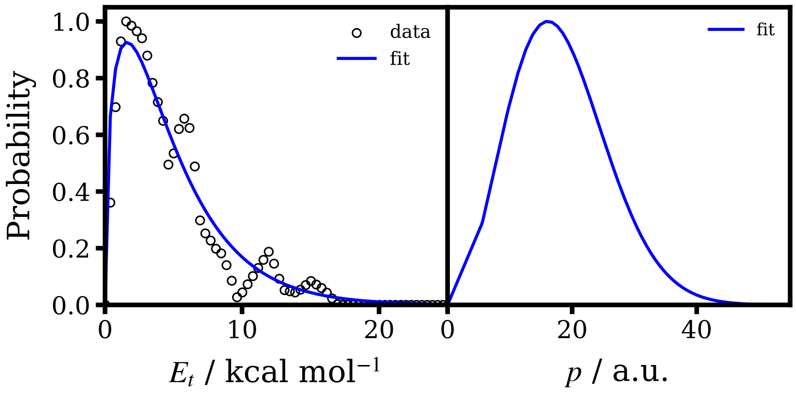

Figure S21: **Asymptotic energy and momentum distributions for $C_6H_5NO^+$.** The left panel shows the probability distribution of total kinetic energy release. Experimental data (black circles) are adapted from Ref. [2], and the fitted distribution is shown in blue. The right panel presents the corresponding fitted function in momentum space.

Since no Coulomb curves were observed for this ion, the time- and momentum-dependent intensity is described solely by Eq. (7). Including channel IV and GSB in the simulation produces Fig. S22.

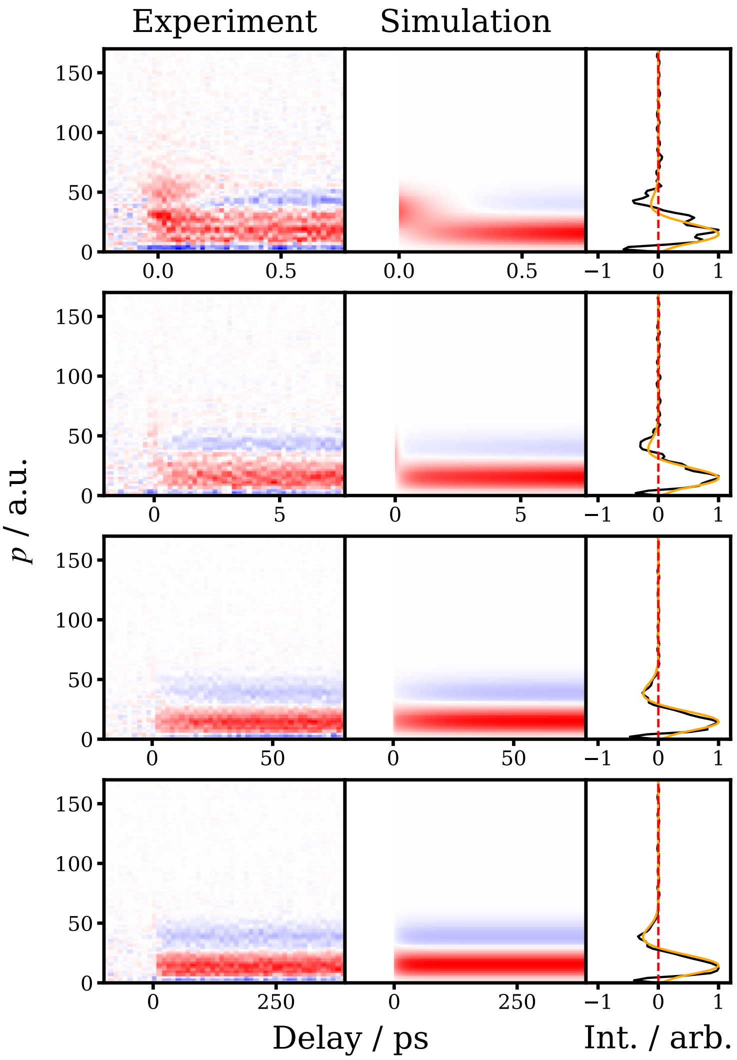

Figure S22: **Comparison of the time-resolved momentum distribution for $C_6H_5NO^+$ with the heuristic simulation incorporating channel IV and the GSB feature.** In the left and middle columns, red and blue represent positive and negative signals, respectively. The right column shows the momentum distribution integrated over the final 10% of the pump-probe delay time, where the black line corresponds to experimental data and the orange line to the simulation.

References

[1] Galloway, D. B., Bartz, J. A., Huey, L. G., and Crim, F. F. *J. Chem. Phys.* **98**, 2107–2114 (1993).

[2] Lin, M., Lee, Y. T., Ni, C., Xu, S., and Lin, M. C. *J. Chem. Phys.* **126**, 064310 (2007).

[3] Garcia, A., Nahon, L., and Powis, I. *Rev. Sci. Instrum.* **75**, 4989–4996 (2004).

[4] McManus, J., Allum, F., Featherstone, J., Lam, C.-S., and Brouard, M. *J. Phys. Chem. A* **128**, 3220 (2024).

[5] Slater, C. S., Blake, S., Brouard, M., Lauer, A., Vallance, C., Bohun, C. S., Christensen, L., Nielsen, J. H., Johansson, M. P., and Stapelfeldt, H. *Phys. Rev. A* **91**, 053424 (2015).

[6] Giussani, A. and Worth, G. A. *J. Chem. Phys.* **157**, 204301 (2022).

[7] Slater, C. S., Blake, S., Brouard, M., Lauer, A., Vallance, C., John, J. J., Turchetta, R., Nomerotski, A., Christensen, L., Nielsen, J. H., Johansson, M. P., and Stapelfeldt, H. *Phys. Rev. A* **89**, 011401 (2014).

[8] Hsieh, S. and Eland, J. H. D. *J. Phys. B: Atom. Mol. Opt. Phys.* **30**, 4515–4534 (1997).

[9] McManus, J. W., Walmsley, T., Nagaya, K., Harries, J. R., Kumagai, Y., Iwayama, H., Ashfold, M. N., Britton, M., Bucksbaum, P. H., Downes-Ward, B., Driver, T., Heathcote, D., Hockett, P., Howard, A. J., Kukk, E., Lee, J. W. L., Liu, Y., Milesevic, D., Minns, R. S., Niozu, A., Niskanen, J., Orr-Ewing, A. J., Owada, S., Rolles, D., Robertson, P. A., Rudenko, A., Ueda, K., Unwin, J., Vallance, C., Burt, M., Brouard, M., Forbes, R., and Allum, F. *Phys. Chem. Chem. Phys.* **24**, 22699–22709 (2022).

[10] Walmsley, T., Allum, F., Harries, J. R., Kumagai, Y., Lim, S., McManus, J., Nagaya, K., Britton, M., Brouard, M., Bucksbaum, P., Fushitani, M., Gabalski, I., Gejo, T., Hockett, P., Howard, A. J., Iwayama, H., Kukk, E., Lam, C.-s., Minns, R. S., Niozu,

A., Nishimuro, S., Niskanen, J., Owada, S., Razmus, W. O., Rolles, D., Somper, J., Ueda, K., Unwin, J., Wada, S.-i., Woodhouse, J. L., Forbes, R., Burt, M., and Warne, E. M. *J. Phys B* **57**, 235101 (2024).

[11] Doughty, B., Koh, C. J., Haber, L. H., and Leone, S. R. *J. Chem. Phys.* **136**(21), 214303–214303 (2012).

[12] Giussani, A. and Worth, G. A. *J. Phys. Chem. Lett.* **15**, 2216–2221 (2024).

[13] Giussani, A. and Worth, G. A. *Phys. Chem. Chem. Phys.* **22**, 15945–15952 (2020).
